# A step-for-step main-group replica of the Fischer carbene synthesis at a borylene carbonyl

**DOI:** 10.1038/s41467-023-36251-3

**Published:** 2023-05-13

**Authors:** Marcel Härterich, Alexander Matler, Rian D. Dewhurst, Andreas Sachs, Kai Oppel, Andreas Stoy, Holger Braunschweig

**Affiliations:** 1 Institute for Inorganic Chemistry, Julius-Maximilians-Universität Würzburg, Am Hubland, 97074 Würzburg, Germany; 2 Institute for Sustainable Chemistry & Catalysis with Boron, Julius-Maximilians-Universität Würzburg, Am Hubland, 97074 Würzburg, Germany

**Keywords:** Chemical bonding, Chemical bonding, Ligands

## Abstract

The Fischer carbene synthesis, involving the conversion of a transition metal (TM)-bound CO ligand to a carbene ligand of the form [=C(OR’)R] (R, R’ = organyl groups), is one of the seminal reactions in the history of organometallic chemistry. Carbonyl complexes of *p*-block elements, of the form [E(CO)_n_] (E = main-group fragment), are much less abundant than their TM cousins; this scarcity and the general instability of low-valent p-block species means that replicating the historical reactions of TM carbonyls is often very difficult. Here we present a step-for-step replica of the Fischer carbene synthesis at a borylene carbonyl involving nucleophilic attack at the carbonyl carbon followed by electrophilic quenching at the resultant acylate oxygen atom. These reactions provide borylene acylates and alkoxy-/silyloxy-substituted alkylideneboranes, main-group analogues of the archetypal transition metal acylate and Fischer carbene families, respectively. When either the incoming electrophile or the boron center has a modest steric profile, the electrophile instead attacks at the boron atom, leading to carbene-stabilized acylboranes – boron analogues of the well-known transition metal acyl complexes. These results constitute faithful main-group replicas of a number of historical organometallic processes and pave the way to further advances in the field of main-group metallomimetics.

## Abstract (web summary)

Replicating the seminal reactions of transition metals with p-block species is often very challenging. Here, the authors present a step-for-step main-group replica of the historical Fischer carbene synthesis, providing bora-Fischer carbene species.

## Introduction

The 1964 synthesis of [(OC)_5_W{=C(OMe)Me}] by E. O. Fischer and A. Maasböl marked the first synthesis of a TM carbene complex^1^. Although the 1973 Nobel Prize in Chemistry to E. O. Fischer and G. Wilkinson was officially awarded for their independent work on sandwich complexes^2^, the fact that Fischer dedicated the entirety of his Nobel Lecture to transition-metal (TM) carbene and carbyne chemistry is a striking testament to the importance of metal-carbon multiple bonding. Thereafter followed decades of major advances in the field, for instance the development of the Fischer / Schrock carbene duality concept^3,4^, the Dötz reaction^5^, and olefin metathesis^6^, to name just a few. The implications of metal-carbon multiple bonding have since suffused further afield into organic chemistry, catalysis, nanoscience^7^ and beyond, and no teaching course on organometallic chemistry is complete without a thorough treatment of the topic.

The ability of main-group compounds to mimic the reactivity of transition metals has been gradually realized over the last 15 years^8–10^. This is mainly due to impressive advances in the synthesis of main-group carbene complexes and low-valent p-block species^11,12^, the latter of which often have TM-like orbital properties. Particularly, the presence of filled and vacant orbitals that are close both in space and energy allows such low-valent main-group ambiphiles to cleave strong bonds of reactants and thus “activate” relatively inert species^13^. This bonding situation can be created both using dual-site ambiphiles (i.e., frustrated Lewis pairs^14^), or single-site ambiphiles (e.g., carbenes, borylenes^15^). A noteworthy achievement in this regard is our binding, reduction (and in some cases also coupling) of dinitrogen mediated by a transient, superambiphilic borylene species^16–18^.

Despite the achievements in the chemistry of these “main-group metallomimetics”, the replication of organometallic reactions by p-block species is mostly limited to the field of small-molecule activation, and many archetypal organometallic processes still have no main-group counterparts. This is likely due to both: (a) the generally difficult synthesis and high reactivity of low-valent p-block species, and (b) limitations of the main-group metallomimetic concept itself. In other words, despite their similarities, main-group elements still have more limited oxidation state and orbital flexibility than TMs. One of the most promising families of boron metallomimetics has been the borylene mono- and dicarbonyls, of the form [L_2-n_RB(CO)_n_] (*n* = 1, 2; L = neutral Lewis base)^19–24^, which have shown the propensity to undergo photodecarbonylation and ligand exchange, both of which are well-established processes of TM carbonyl complexes.

**Fig. 1:**
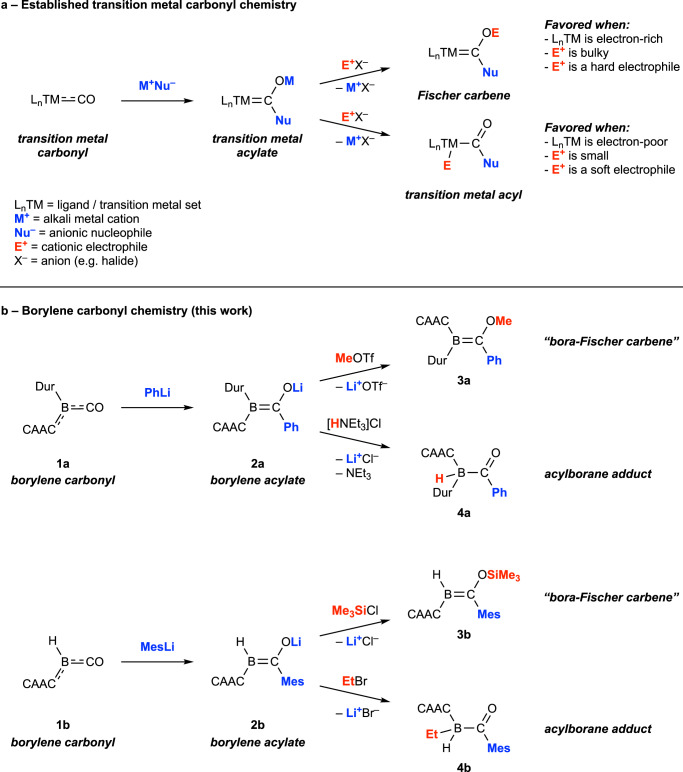
Traditional chemistry of transition-metal carbonyls with nucleophiles and main-group metallomimetic analogues based on boron. **a** Fischer carbene synthesis and a variant thereof. Strong nucleophiles generally attack TM carbonyls at the carbon atom of a carbonyl ligand, leading to TM acylate complexes, which, when treated a cationic electrophile, provide either a Fischer carbene complex or a TM acyl complex, depending on a range of factors. **b** Fischer carbene synthesis at boron. The Fischer carbene synthesis can by replicated with metallomimetic borylene carbonyls, leading to bora-Fischer carbenes or acylborane Lewis adducts, main-group analogues of the traditional TM counterparts.

These reactions prompted us to question whether borylene carbonyls could serve as metallomimetic scaffolds for replicating other classical reactivity patterns of TM carbonyl complexes. We envisaged that a carbene-stabilized borylene carbonyl ([LRB(CO)], L = carbene donor) might allow us to demonstrate a main-group analogue of the Fischer carbene synthesis protocol (Fig. 1a). A further promising sign was our recent discovery that, under reducing conditions, a borylene carbonyl undergoes intramolecular migration of an aryl group to its carbonyl carbon atom, decomposing the carbene unit but furnishing a “bora-Fischer carbene” species^23^. In this work, we present the controlled nucleophilic attack at the carbonyl carbon atom of a borylene carbonyl, followed by quenching of the resulting borylene acylate with an electrophile, leading to alkoxy-/silyloxy-substituted alkylideneboranes. Varying the electrophile in certain cases leads to attack at the boron atom and the isolation of carbene adducts of acylboranes – boron analogues of the well-known transition-metal acyl complexes. Together, the reactions demonstrate main-group analogues of three of the key elemental reactions in the chemistry of TM carbonyl chemistry.

## Results and discussion

### Step one: nucleophilic attack at a carbonyl ligand

**Fig. 2:**
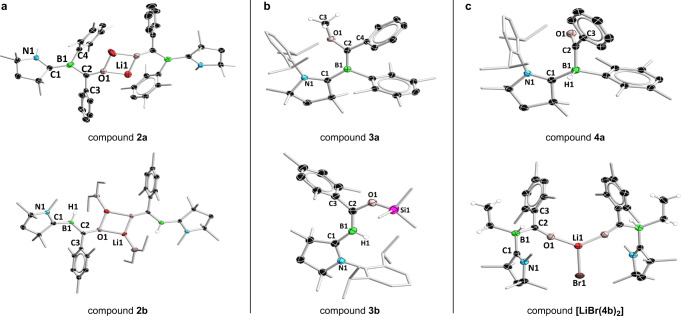
Crystallographically derived molecular structures of products. **a** borylene acylates (aryl groups of the CAAC units truncated for clarity). **b** bora-Fischer carbenes. **c** acylborane Lewis adducts (in the case of **4b**, the LiBr coordination complex of two acylborane adducts, **[LiBr(4b)**_**2**_**]**).

The first step of the historical Fischer carbene synthesis^1,2^ is the nucleophilic attack at the carbon atom of a metal-bound CO ligand, forming an anionic complex with an acylate ligand ([L_n_M(C(O)R)]^–^) (Fig. 1a). This step is essentially a variant of the first step of another foundational organometallic reaction from 1931, the Hieber base reaction, in which a hydroxide anion (OH^–^) attacks at the carbon atom of [Fe(CO)_5_], forming the anionic acylate complex [(OC)_5_Fe–C(O)OH]^–^^25^. Our attempts to recreate this reaction centered on two borylene carbonyls stabilized by a cyclic (alkyl)(amino)carbene (CAAC; 1-(2,6-diisopropylphenyl)−3,3,5,5-tetramethylpyrrolidin-2-ylidene), namely [(CAAC)BDur(CO)] (**1a**; Dur = 2,3,5,6-tetramethylphenyl) and [(CAAC)BH(CO)] (**1b**, present as a ca. 1:1 mixture of *E*/*Z* isomers^24^. Treatment of **1a,b** with the aryl nucleophiles phenyllithium and mesityllithium, respectively, provided highly reactive red solids **2a,b** (Fig. 1b). Complex **2a** could only be characterized by its ^11^B NMR spectrum and a single-crystal X-ray diffraction (SCXRD) study that provided poor data suitable for establishment of its connectivity. However, complex **2b** was fully characterized by NMR spectroscopy (^1^H, ^7^Li, ^11^B, ^13^C), high-resolution mass spectrometry (HRMS) and SCXRD. Their solid-state structures indicated that complexes **2a,b** were indeed the desired borylene acylate species resulting from the nucleophilic attack at the positively charged boron atom of complexes **1a,b** (for ADCH of **1b** partial charges see Supplementary Fig. 28), which crystallize as their lithium-bound dimers (Fig. 2a). In both compounds, the OCBCN segments are only slightly distorted from planar and suggest a high degree of π-delocalization in this unit. This delocalization is reflected in the fact that the distances between the boron atom and its attached carbon atoms are very similar (**2a**: B–C^CAAC^ 1.510(8), 1.496(9) Å; B–C^acyl^ 1.520(7), 1.532(8) Å; **2b**: B–C^CAAC^ 1.507(6), 1.508(6) Å; B–C^acyl^ 1.502(5), 1.494(5) Å). Resonances in the ^11^B NMR spectra of **2a,b** were found at relatively high field (**2a**: 24.0 ppm; **2b**: 10.8 ppm), suggesting relatively high electron density at the boron atom. However, these are still significantly downfield of the resonances of their respective precursor borylene carbonyls (**1a**: −13.4 ppm^21^; **1b**: –23.9, –24.5 ppm^24^), which are electron-rich, low-valent boron species. To our knowledge, only one other such borylene acylate species is known in the literature, a potassium-bound dimer reported by our group, which is generated by an unusual reduction / aryl migration process from a borylene carbonyl^23^. This compound has an analogous structure to **2a,b**, with a delocalized OCBCN unit, B–C^CAAC^ and B–C^acyl^ distances of 1.503(2) and 1.518(2) Å, and an ^11^B NMR resonance at 15.0 ppm.

### Step two: electrophilic quench of borylene acylates

The second step of the Fischer carbene synthesis is the electrophilic quenching of the anionic acylate complex of the form [L_n_M(C(O)R)]^–^, whereby the electrophile (E^+^) adds at the acylate oxygen atom, furnishing the Fischer carbene species [L_n_M = C(OE)R]^2^. Alternatively, attack of the electrophile at the metal center may take place, providing an acyl complex (Fig. 1a) that inevitably undergoes reductive elimination of the –E and –C(O)R ligands as an aldehyde (E = H) or ketone (E = organyl group)^26,27^. Of these two options, the Fischer carbene pathway is generally favored when: (a) the metal center is relatively electron poor (i.e., the metal does not compete with the acylate oxygen in terms of nucleophilicity), (b) the electrophile is sterically bulky and may struggle to approach the crowded metal site, and/or (c) the electrophile is relatively “hard” and thus a good match for an acylate oxygen nucleophile.

Astonishingly, for each of the two borylene acylate species **2a,b**, both the reaction pathways described above (electrophilic attack at acylate oxygen or “metal”) were found to be possible by employing different electrophiles. Treatment of **2a** with methyl triflate, and **2b** with trimethylsilyl chloride, led to isolation of **3a** (53% yield) and **3b** (82% yield), respectively, as red solids (Fig. 1b). Their ^11^B NMR spectroscopic resonances (**3a**: 19.0 ppm; **3b**: 15.3 ppm) are found in a similar region to those of their acylate precursors, the former having moved slightly upfield and the latter slightly downfield; likely a consequence of the disparate nature of the two electrophiles (i.e., carbon- vs. silicon-centered). In their solid-state structures (Fig. 2b) the two B–C bonds of the OCBCN units of **3a,b** are much more distinct in length (**3a**: B–C^CAAC^ 1.551(3), 1.560(3) Å; B=C(OE)R 1.487(3), 1.492(3) Å; **3b**: B–C^CAAC^ 1.532(2) Å; B=C(OE)R 1.467(2) Å) than those of the acylate precursors **2a,b**, indicating greater B=C(OE)R double-bond character and suggesting that they are best described as CAAC-stabilized alkoxy-/silyloxyalkylideneboranes, or alternatively “bora-Fischer carbenes”. These spectroscopic and structural data are in line with those of the three previously reported CAAC-stabilized alkylideneboranes bearing hydroxy, alkoxy or silyloxy groups at the alkylidene carbon atom^23^, the ^11^B NMR resonances of which are in the range 15–18 ppm, and which also feature more B=C(OE)R double-bond character (B=C(OE)R 1.47–1.48 Å).

**Fig. 3:**
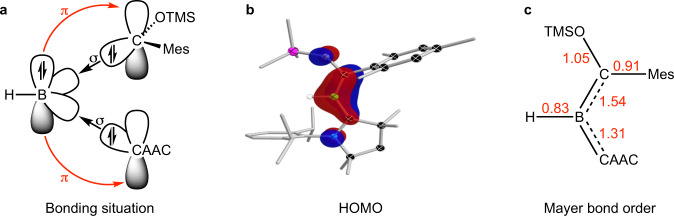
Description of the bonding situation of compound 3b (level of theory: B3-LYP/def2-SVP with GD3 dispersion correction). **a** relevant orbital interactions for bond formation. **b** HOMO of the bora-Fischer carbene. **c** Mayer bond orders in the CAAC-stabilized alkylideneborane.

The presence of both Fischer-type and heterocyclic carbene units bound to boron in the complexes provides a rare opportunity to directly compare these two ligand types^28^. Based on DFT calculations, the nature of the bonding in compounds **3a,b** can be described according to Fig. 3, whereby both carbenes are σ-donors to the boron, and the boron atom in turn provides π backdonation. The Mayer bond orders (MBOs) within compound **3b** suggest that there is strong π delocalization in the CBC unit, but that the B=C^Fischer^ bond (MBO: 1.31) is slightly stronger than the B=C^CAAC^ bond (MBO: 1.31). Similarly, the HOMO comprises the CBC π interaction and suggests that the B=C π bonding is slightly stronger to the Fischer carbene than the CAAC unit.

Protonation of in-situ-generated acylate complex **2a** with triethylammonium chloride ([HNEt_3_]Cl) provided acylborane adduct **4a** as an orange solid (36% yield), while treatment of **2b** with ethyl bromide provided the analogous **4b** as its pale yellow complex with lithium bromide, [LiBr(**4b**)_2_] (82% yield) (Figs. 1b, 2c). Attempts to sequester the LiBr by dissolving the compound in non-polar solvents such as benzene and hexane in order to obtain pure **4b** were unsuccessful. Both acylborane adducts showed doublet resonances in their ^11^B NMR spectra (**4a**: –14.8 ppm, ^1^*J*_BH_ = 77.2 Hz; [LiBr(**4b**)_2_]: –12.7 ppm, ^2^J_BH_ = 84.6 Hz) thanks to coupling with their boron-bound hydrides. While acylboranes (RC(O)BR'_2_) and acylborates ([RC(O)BR'_3_]^–^) are well known and synthetically useful species^29,30^, to our knowledge, structurally authenticated singly base-stabilized acylboranes (RC(O)BR'_2_L; R = organyl group) such as **4a,b** are unknown.

The divergent reactions of the borylene acylates **2a,b** with different electrophiles invites analysis in light of the determinants for electrophilic attack at either the metal or acylate oxygen of TM acylate complexes, as outlined in Fig. 1a (right side)^26,27^. Given that both variants occur with both borylene ([(CAAC)BR]) scaffolds, the electron density of the borylene unit itself is likely not a determinant of the reaction pathway for these compounds. This is borne out by the very similar calculated atomic dipole-moment-corrected Hirshfeld (ADCH) charges of the boron and acylate oxygen atoms of **2b** (B: −0.241; O: −0.331). In comparison, the negative charge of the tungsten acylate complex used in the original Fischer carbene synthesis ([(OC)_5_M(C(O)Ph)]^–^) is much more concentrated at the acylate O atom than the W atom (W: −0.068; O: −0.411). The softness/hardness of the electrophile can also be ruled out as a factor given that there is no clear pattern of matching soft or hard electrophiles and nucleophiles in the reactions shown in Fig. 1b (e.g., the alkyl electrophile attacks at the acyl oxygen of **2a** but the boron atom of **2b**, etc.). Therefore, the electrophilic quenching reactions in Fig. 1b appear to be under steric control, as for each borylene acylate the smaller electrophile attacks at the far more crowded boron site while the larger electrophile attacks at the less crowded oxygen site. It should be noted that many more electrophilic quench reactions were attempted with a variety of electrophiles; however, only those shown in Fig. 1b led to tractable products, preventing us from obtaining a more general overview of the factors underpinning the regioselectivity. In addition, in each quench reaction shown, the presented product (either of type **3** or **4**) was the sole product in the reaction mixture at full consumption of the precursor, and the alternative isomer was not detected.

In conclusion, we present herein a step-for-step main-group analogue of the Fischer carbene synthesis, resulting in carbene-stabilized alkoxy-/silyloxy-substituted alkylideneboranes, which can be considered boron analogues of conventional, transition-metal Fischer carbene complexes. Alternatively, by varying the electrophilic reagent in the synthesis, boron analogues of archetypal TM acyl complexes can be prepared. These reactions extend the use of p-block species as mimics of transition metals beyond small-molecule activation by replicating multi-step syntheses that are of historical and pedagogical importance in traditional, transition-metal-based organometallic chemistry. This work also opens up diverse opportunities in developing further metallomimetic chemistry using low-valent boron species, in particular replicating well-known reactions of TM Fischer carbenes, e.g., the Dötz reaction, olefin metathesis, or the synthesis of main-group analogues of Fischer carbynes. Work in our laboratories is underway with these goals in mind.

## Methods

### General synthetic considerations

All manipulations were performed either under an atmosphere of dry argon or in vacuo using standard Schlenk line or glovebox techniques. Deuterated solvents were dried over molecular sieves and degassed by three freeze-pump-thaw cycles prior to use. All other solvents were distilled and degassed from appropriate drying agents. Solvents were stored under argon over activated 4 Å molecular sieves. NMR spectra were acquired on a Bruker Avance 400 or 500 NMR spectrometer. Chemical shifts (*δ*) are provided in ppm and internally referenced to the carbon nuclei (^13^C{^1^H}) or residual protons (^1^H) of the solvent. Heteronuclei NMR spectra are referenced to external standards (^11^B: BF_3_$\bullet$OEt_2_). Solid-state IR spectra were recorded on a Bruker FT-IR spectrometer ALPHA II inside a glovebox. UV-vis spectra were measured on a METTLER TOLEDO UV-vis-Excellence UV5 spectrophotometer inside a glovebox. Microanalyses (C, H, N) were performed on an Elementar vario MICRO cube elemental analyzer. High-resolution mass spectrometry data were obtained from a Thermo Scientific Exactive Plus spectrometer in LIFDI or ASAP mode. Syntheses of known compounds following published procedures can be found in the Supplementary methods section of the Supplementary Data file, with accompanying Supplementary Figs. 29–33.

### Synthesis of new compounds

#### Synthesis of [(CAAC)B(Dur)=C(Ph)(OMe)] (3a)

[(CAAC)BDur(CO)] (**1a**) (100 mg, 219 µmol) was dissolved in 20 mL of benzene and phenyllithium (40.4 mg, 481 µmol) was added. The reaction mixture was stirred at room temperature for 3 h and methyltrifluoromethanesulfonate (79.3 mg, 483 µmol) was added. After 2 h of stirring at room temperature all volatiles were removed in vacuo. The solid residue was extracted with hexane and recrystallized at –30 °C, yielding [(CAAC)B(Dur)=C(Ph)(OMe)] (**3a**) as a red solid in 53% yield (63.8 mg, 116 µmol). ^1^H NMR (500.1 MHz, 298 K, C_6_D_6_): δ = 7.08 (s, 3H, Dip-C*H*), 6.83–6.89 (m, 4H, Ph-C*H*), 6.78 (s, 1H, Dur-C*H*), 6.75–6.72 (m, 1H, *p*-Ph-C*H*), 3.24 (sept, ^3^*J*_HH_ = 6.6 Hz, 2H, C*H*(CH_3_)_2_), 2.47 (s, 6H, *o*-Dur-C*H*_3_), 2.42 (s, 3H, OC*H*_3_), 2.17 (s, 6H, *m*-Dur-C*H*_3_), 1.67–1.59 (m, 6H, CH(C*H*_3_)_2_), 1.33 (d, ^3^*J*_HH_ = 6.6 Hz, 6H, CH(C*H*_3_)_2_), 1.25 (s, 6H, CC(C*H*_3_)_2_), 1.02 (s, 6H, NC(C*H*_3_)_2_) ppm. ^13^C NMR (125.8 MHz, 298 K, C_6_D_6_) (BC_q_O could not be detected): δ = 194.6 (*C*_carbene_), 146.4 (*i*-Dip-*C*_q_), 144.1 (Ph-*C*_q_), 138.8 (*o*-Dip-*C*_q_), 138.3 (*m*-Dur-*C*_q_), 131.8 (*o*-Dur-*C*_q_), 129.4 (Dur-*C*H), 129.1 (*m*-Ph-*C*H), 128.1 (Dip-*C*H), 126.1 (*o*-Ph-*C*H), 125.2 (*p*-Ph-*C*H), 124.0 (Dip-*C*H), 72.8 (N*C*(CH_3_)_2_), 57.1 (O*C*H_3_), 55.3 (*C*H_2_), 51.6 (C*C*(CH_3_)_2_), 30.3 (CC(*C*H_3_)_2_), 29.8 (*C*H(CH_3_)_2_), 29.3 (NC(*C*H_3_)_2_), 26.5 (CH(*C*H_3_)_2_), 24.5 (CH(*C*H_3_)_2_), 23.0 (*o*-Dur-*C*H_3_), 21.0 (*m*-Dur-*C*H_3_) ppm. ^11^B NMR (160.5 MHz, 298 K, C_6_D_6_): δ = 19.0 (s) ppm. LIFDI-HRMS calculated for [C_36_H_52_BNO] = [M]^+^ 549.4136; found: 549.4141.

#### Synthesis of [(CAAC)B(Dur)(H)C(=O)(Ph)] (4a)

[(CAAC)BDur(CO)] (**1a**) (30 mg, 65.6 µmol) was dissolved in 0.5 mL of benzene and phenyllithium (12.1 mg, 144 µmol) was added. The reaction mixture was left at room temperature for 3 h and triethylammonium chloride (20.1 mg, 146 µmol) was added. After 2 h at room temperature all volatiles were removed in vacuo. The solid residue was washed with hexane (3 ×1 mL) and recrystallized at –30 °C from THF, yielding [(CAAC)B(Dur)(H)C(=O)(Ph)] (**4a**) as an orange solid in 36% yield (12.6 mg, 23.6 µmol). ^1^H NMR (400.6 MHz, 298 K, CD_2_Cl_2_): δ = 7.37 (d, ^3^*J*_HH_ = 7.7 Hz 1H, *o*-Ph-C*H*), 7.32 (t, ^3^*J*_HH_ = 7.7 Hz 1H, *p*-Dip-C*H*), 7.20 (d, ^3^*J*_HH_ = 7.7 Hz 1H, *m*-Ph-C*H*), 7.11–7.07 (m, 1H, *p*-Ph-C*H*), 7.02 (t, ^3^*J*_HH_ = 7.5 Hz 2H, *m*-Dip-C*H*), 6.97 (d, ^3^*J*_HH_ = 7.5 Hz 1H, *o*-Ph-C*H*), 6.66 (s, 1H, Dur-C*H*), 4.06 (q, ^1^*J*_BH_ = 74.84 Hz, B*H*), 3.10 (sept., ^3^*J*_HH_ = 6.8 Hz 1H, C*H*(CH_3_)_2_), 2.63 (sept., ^3^*J*_HH_ = 6.3 Hz, 1H, C*H*(CH_3_)_2_), 2.43 (s (br), 6H, *o*-Dur-C*H*_3_), 2.08 (s (br), 6H, *m*-Dur-C*H*_3_), 2.07 (d, ^3^*J*_HH_ = 12.8 Hz 1H, C*H*_2_), 1.99 (d, ^3^*J*_HH_ = 12.8 Hz 1H, C*H*_2_), 1.78 (d, ^3^*J*_HH_ = 6.3 Hz, 3H, CH(C*H*_3_)_2_), 1.64 (s, 3H, NC(C*H*_3_)_2_), 1.43 (s, 3H, CC(C*H*_3_)_2_), 1.37 (d, ^3^*J*_HH_ = 6.4 Hz, 3H, CH(C*H*_3_)_2_), 1.28 (s, 3H, NC(C*H*_3_)_2_), 1.19 (d, ^3^*J*_HH_ = 6.8 Hz, 3H, CH(C*H*_3_)_2_), 1.18 (s, 3H, CC(C*H*_3_)_2_), 1.03 (d, ^3^*J*_HH_ = 6.5 Hz, 3H, CH(C*H*_3_)_2_) ppm. ^13^C NMR (100.7 MHz, 298 K, C_6_D_6_) (BC_q_O could not be detected): δ = 146.7 (*o*-Dip-*C*_q_), 145.1 (*i*-Dip-*C*_q_), 139.9 (*m*-Dur-C_q_), 138.6 (*o*-Dur-C_q_) 136.5 (*i*-Ph-*C*_q_), 129.3 (Dur-*C*H), 129.2 (*p*-Dip-*C*H), 129.1 (*p*-Ph-*C*H), 127.1 (*m*-Ph-*C*H), 127.1 (*m*-Dip-*C*H), 125.7 (*o*-Ph-*C*H), 125.2 (*o*-Ph-*C*H), 77.9 (N*C*(CH_3_)_2_), 54.8 (C*C*(CH_3_)_2_), 54.7 (*C*H_2_), 30.8 (CC(*C*H_3_)_2_), 30.3 (NC(*C*H_3_)_2_), 30.2 (CH(*C*H_3_)_2_), 29.2 (CH(*C*H_3_)_2_), 29.0 (NC(*C*H_3_)_2_), 28.4 (CH(*C*H_3_)_2_), 27.5 (CH(*C*H_3_)_2_), 26.4 (CC(*C*H_3_)_2_), 25.4 (CH(*C*H_3_)_2_), 25.1 (CH(*C*H_3_)_2_), 22.5 (*o*-Dur-C*H*_3_), 21.3 (*m*-Dur-C*H*_3_) ppm. ^11^B NMR (128.5 MHz, 298 K, C_6_D_6_): δ = –14.8 (d, ^1^*J*_BH_ = 77.2 Hz, B*H*) ppm. LIFDI-HRMS calculated for [C_36_H_52_BNO] = [M]^+^ 549.4136; found: 549.4141.

#### Synthesis of [(CAAC)B(H)=C(Mes)(OLi(OEt_2_))] (2b)

[(CAAC)BH(CO)] (**1b**) (20 mg, 61.5 µmol) and mesityllithium etherate (16 mg, 80.0 µmol) were suspended in diethyl ether (0.7 mL). After 1 h at room temperature the reaction mixture was filtered and crystallized at –30 °C, yielding [(CAAC)B(H)=C(Mes)(OLi(OEt_2_))] (**2b**) as a red solid in 96% yield (31 mg, 58.9 µmol). ^1^H NMR (500.1 MHz, 298 K, C_6_D_6_): δ = 6.98 (dd, 1H, ^3^*J*_HH_ = 7.5 Hz, ^3^*J*_HH_ = 7.5 Hz, *p*-Dip-C*H*), 6.94 (d, 2H, ^3^*J*_HH_ = 7.5 Hz, *m*-Dip-C*H*), 6.44 (s, 2H, Mes-C*H*), 3.38 (q, 4H, ^3^*J*_HH_ = 7.0 Hz, Et_2_O-C*H*_2_), 3.22 (sept, 2H, ^3^*J*_HH_ = 6.8 Hz, C*H*(CH_3_)_2_), 2.63 (br, 1H, B*H*), 2.16 (s, 6H, *o*-Mes-C*H*_3_), 2.05 (s, 3H, *p*-Mes-C*H*_3_), 1.95 (s, 2H, C*H*_2_), 1.83 (s, 6H, CC(C*H*_3_)_2_), 1.18 (d, 6H, ^3^*J*_HH_ = 6.8 Hz, CH(C*H*_3_)_2_), 1.15 (d, 6H, ^3^*J*_HH_ = 6.7 Hz, CH(C*H*_3_)_2_), 1.12 (s, 6H, NC(C*H*_3_)_2_), 1.12 (t, 6H, ^3^*J*_HH_ = 7.0 Hz, Et_2_O-C*H*_3_) ppm. ^13^C NMR (125.8 MHz, 298 K, C_6_D_6_) (BC_q_O could not be detected): δ = 182.3 (C_Carbene_), 155.4 (*i*-Mes-*C*_q_), 150.0 (*i*-Dip-*C*_q_), 139.4 (*o*-Dip-*C*_q_), 132.9 (*o*-Mes-*C*_q_), 131.6 (*p*-Mes-*C*_q_), 127.5 (Mes-*C*H), 126.6 (*p*-Dip-*C*H), 123.8 (*m*-Dip-*C*H), 66.3 (Et_2_O-*C*H_2_), 66.3 (N*C*(CH_3_)_2_), 58.8 (*C*H_2_), 45.9 (C*C*(CH_3_)_2_), 31.0 (CC(*C*H_3_)_2_), 30.3 (NC(*C*H_3_)_2_), 29.1 (*C*H(CH_3_)_2_), 27.2 (CH(*C*H_3_)_2_), 24.2 (CH(*C*H_3_)_2_), 21.0 (*p*-Mes-*C*H_3_), 20.2 (*o*-Mes-*C*H_3_), 15.7 (Et_2_O-*C*H_3_) ppm. ^7^Li NMR (194.4 MHz, 298 K, C_6_D_6_): δ = 0.2 (s) ppm. ^11^B NMR (160.5 MHz, 298 K, C_6_D_6_): δ = 10.8 (s) ppm. LIFDI-HRMS calculated for [C_30_H_44_BNO] = [M-Et_2_O-Li]^+^ 445.3510; found: 445.3508.

#### Synthesis of [(CAAC)B(H)=C(Mes)(OSiMe_3_)] (3b)

[(CAAC)BH(CO)] (**1b**) (20 mg, 61.5 µmol) and mesityllithium etherate (16 mg, 80.0 µmol) were suspended in diethyl ether (0.7 mL). The reaction mixture was left at room temperature for 20 min and chlorotrimethylsilane (60 mg, 552 µmol) was added. After 1 h at room temperature all volatiles were removed *in vacuo*. The solid residue was extracted with hexane and recrystallized, yielding [(CAAC)B(H)=C(Mes)(OSiMe_3_)] (**3b**) as a orange solid in 82% yield (26 mg, 50.2 µmol). ^1^H NMR (500.1 MHz, 298 K, C_6_D_6_): δ = 7.22 (dd, 1H, ^3^*J*_HH_ = 6.7 Hz, ^3^*J*_HH_ = 8.6 Hz, *p*-Dip-C*H*), 7.16 (br d (coincides with solvent), 1H, *m*-Dip-C*H*), 7.14 (br d, ^3^*J*_HH_ = 8.5 Hz, 1H, *m*-Dip-C*H*), 6.90 (s, 2H, Mes-C*H*), 3.50 (br, 1H, B*H*), 2.88 (sept, 2H, ^3^*J*_HH_ = 6.7 Hz, C*H*(CH_3_)_2_), 2.60 (s, 6H, *o*-Mes-C*H*_3_), 2.20 (s, 3H, *p*-Mes-C*H*_3_), 1.52 (s, 2H, C*H*_2_), 1.50 (d, 6H, ^3^*J*_HH_ = 6.7 Hz, CH(C*H*_3_)_2_), 1.21 (d, 6H, ^3^*J*_HH_ = 6.7 Hz, CH(C*H*_3_)_2_), 1.19 (s, 6H, CC(C*H*_3_)_2_), 0.95 (s, 6H, NC(C*H*_3_)_2_), 0.22 (s, 9H, Si(C*H*_3_)_3_) ppm. ^13^C NMR (125.8 MHz, 298 K, C_6_D_6_): δ = 207.7 (*C*_carbene_), 181.5 (BC_q_O), 146.6 (*i*-Dip-*C*_q_), 145.1 (*i*-Mes-*C*_q_), 136.7 (*o*-Mes-*C*_q_), 135.9 (*o*-Dip-*C*_q_), 135.7 (*p*-Mes-*C*_q_), 128.7 (*p*-Dip-*C*H), 128.5 (Mes-*C*H), 124.7 (*m*-Dip-*C*H), 70.9 (N*C*(CH_3_)_2_), 55.4 (*C*H_2_), 48.5 (C*C*(CH_3_)_2_), 29.8 (CC(*C*H_3_)_2_), 29.3 (*C*H(CH_3_)_2_), 28.9 (NC(*C*H_3_)_2_), 26.3 (CH(*C*H_3_)_2_), 23.9 (CH(*C*H_3_)_2_), 21.3 (*p*-Mes-*C*H_3_), 21.1 (*o*-Mes-*C*H_3_), 1.9 (Si(*C*H_3_)_3_) ppm. ^11^B NMR (160.5 MHz, 298 K, C_6_D_6_): δ = 15.3 (s) ppm. ^29^Si NMR (99.4 MHz, 298 K, C_6_D_6_): δ = 10.0 (s) ppm. LIFDI-HRMS calculated for [C_33_H_52_BNOSi] = [M]^+^ 517.3906; found: 517.3904.

#### Synthesis of [((CAAC)B(H)(Et)C(=O)(Mes))_2_LiBr] ([LiBr(4b)_2_])

[(CAAC)BHCO] (**1b**) (20 mg, 61.5 µmol) and mesityllithium etherate (16 mg, 80.0 µmol) were suspended in diethyl ether (0.7 mL). The reaction mixture was left at room temperature for 20 min and ethyl bromide (20 mg, 185 µmol) was added. After 2 d at 60 °C all volatiles were removed *in vacuo*. The solid residue was extracted with hexane and recrystallized. After washing with hexane (2 ×1 mL) [((CAAC)B(H)(Et)C(=O)(Mes))_2_LiBr] (**[LiBr(4b)**_**2**_**]**) was obtained as a pale yellow solid in 82% yield (26 mg, 50.3 µmol). ^1^H NMR (500.1 MHz, 298 K, *d*_*8*_-THF): δ = 7.37 (dd, 1H, ^3^*J*_HH_ = 7.9 Hz, ^3^*J*_HH_ = 7.9 Hz, *p*-Dip-C*H*), 7.29 (dd, 1H, ^3^*J*_HH_ = 8.1 Hz, ^4^*J*_HH_ = 1.8 Hz, *m*-Dip-C*H*), 7.28 (dd, 1H, ^3^*J*_HH_ = 8.1 Hz, ^4^*J*_HH_ = 1.8 Hz, *m*-Dip-C*H*), 6.54 (s, 2H, Mes-C*H*), 3.23 (sept, 1H, ^3^*J*_HH_ = 6.5 Hz, C*H*(CH_3_)_2_), 2.66 (sept, 1H, ^3^*J*_HH_ = 6.6 Hz, C*H*(CH_3_)_2_), 2.19 (d, 1H, ^2^*J*_HH_ = 12.7 Hz, CC*H*_2_), 2.12 (s, 3H, *p*-Mes-C*H*_3_), 2.08 (br, 1H, B*H*), 2.05 (d, 1H, ^2^*J*_HH_ = 12.7 Hz, CC*H*_2_), 2.00 (s, 6H, *o*-Mes-C*H*_3_), 1.66 (s, 3H, CC(C*H*_3_)_2_), 1.53 (s, 3H, CC(C*H*_3_)_2_), 1.43 (s, 3H, NC(C*H*_3_)_2_), 1.34 (s, 3H, NC(C*H*_3_)_2_), 1.30 (d, 3H, ^3^*J*_HH_ = 6.5 Hz, CH(C*H*_3_)_2_), 1.29 (d, 3H, ^3^*J*_HH_ = 6.5 Hz, CH(C*H*_3_)_2_), 1.23 (d, 3H, ^3^*J*_HH_ = 6.5 Hz, CH(C*H*_3_)_2_), 1.04 (d, 3H, ^3^*J*_HH_ = 6.5 Hz, CH(C*H*_3_)_2_), 0.72–0.62 (m, 1H, BC*H*_2_), 0.50–0.39 (m, 1H, BC*H*_2_), 0.44 (s, 3H, BCH_2_C*H*_3_) ppm. ^13^C NMR (125.8 MHz, 298 K, *d*_*8*_-THF) (the signal corresponding to the BC_q_O nucleus could not be detected): δ = 238.0 (*C*_carbene_), 149.7 (*i*-Mes-*C*_q_), 147.0 (*o*-Dip-*C*_q_), 145.8 (*o*-Dip-*C*_q_), 135.2 (*p*-Mes-*C*_q_), 134.7 (*i*-Dip-*C*_q_), 133.0 (*o*-Mes-*C*_q_), 129.6 (*p*-Dip-*C*H), 128.8 (Mes-*C*H), 125.9 (*m*-Dip-*C*H), 125.2 (*m*-Dip-*C*H), 79.0 (N*C*(CH_3_)_2_), 54.3 (C*C*(CH_3_)_2_), 52.6 (*C*H_2_), 30.7 (NC(*C*H_3_)_2_), 30.1 (CC(*C*H_3_)_2_), 29.9 (CC(*C*H_3_)_2_), 29.7 (*C*H(CH_3_)_2_), 29.5 (*C*H(CH_3_)_2_), 28.9 (CH(*C*H_3_)_2_), 28.4 (NC(*C*H_3_)_2_), 25.7 (CH(*C*H_3_)_2_), 25.3 (CH(*C*H_3_)_2_), 24.4 (CH(*C*H_3_)_2_), 21.1 (*o*-Mes-*C*H_3_), 20.8 (*p*-Mes-*C*H_3_), 14.3 (BCH_2_*C*H_3_), 12.0 (B*C*H_2_CH_3_) ppm. ^7^Li NMR (194.4 MHz, 298 K, *d*_*8*_-THF): δ = 0.7 (s) ppm. ^11^B NMR (160.5 MHz, 298 K, *d*_*8*_-THF): δ = –12.7 (d, ^2^J_BH_ = 84.6 Hz, *B*H) ppm. LIFDI-HRMS calculated for [C_32_H_48_BNO] = [M]^+^ 473.3823; found: 473.3823.

### Single-crystal X-ray diffraction

#### General considerations

The crystal data were collected either on a Bruker D8 Quest diffractometer with a CMOS area detector ([(CAAC)B(Dur)=C(Ph)(OMe)] (**3a**), [(CAAC)B(H)(Dur)C(=O)(Ph)] (**4a**), [(CAAC)B(H)=C(Mes)(OLi(OEt_2_))] (**2b**)) a Bruker X8-APEX II diffractometer with a CCD area detector ([(CAAC)B(Dur)=C(Ph)(OLi)] (**2a**)), both equipped with multi-layer mirror monochromated Mo_κα_ radiation, or a Rigaku OD XtaLAB Synergy-R diffractometer with a HPA area detector, equipped with multi-layer mirror monochromated Cu_κα_ radiation ([(CAAC)B(H)=C(Mes)(OSiMe_3_)] (**3b**) and [((CAAC)B(H)(Et)C(=O)(Mes))_2_LiBr] (**4b**)). The structures were solved using the intrinsic phasing method^31^, refined with the ShelXL program^32^ and expanded using Fourier techniques. All non-hydrogen atoms were refined anisotropically. Hydrogen atoms were included in structure factor calculations. All hydrogen atoms were assigned to idealized positions, except the boron-bound hydrogens, which were detected in the difference Fourier map and freely refined, except when stated otherwise in the refinement details.

### Computational details

Geometry optimizations were performed in the gas phase for **2b** and Li[(OC)_5_W(C(O)Ph)] using the B3LYP^33–36^ functional with the D3^37^ dispersion correction combined with the def2-SVP^38^ basis set. All optimized structures were verified as minimum through vibrational frequency calculations, showing no imaginary frequencies. The electronic situation of the compounds was investigated using ADCH^39^ charges. To support the obtained results for compound **2b**, CHelpG^40^ charges were also calculated and are found to be in good agreement. For Li[(OC)_5_W(C(O)Ph)], only ADCH was applied. All geometry optimizations and frequency calculations were performed with the Gaussian 16, Revision C.01, program^41^. The ADCH and CHelpG charges as well as the Mayer bond orders were obtained using Multiwfn 3.8 program^42^.

## Supplementary information

Supplementary Information

## Data Availability

The NMR and computational data generated in this study are provided in the Supplementary Information file. The crystallographic data used in this study are available in the Cambridge Crystallographic Database under accession codes CCDC 2212592 (**2a**), 2212593 (**2b**), 2212594 (**3a**), 2212591 (**3b**), 2212589 (**4a**), and 2212590 (**[LiBr(4b)**_**2**_**]**). Further data supporting the findings of this study are available from the corresponding author upon request.
