# Supplementary material for: A step-for-step main-group replica of the Fischer carbene synthesis at a borylene carbonyl
Source: Nat Commun. 2023 May 13;14:2764. doi: 10.1038/s41467-023-36251-3 (PMC10183005; doi:10.1038/s41467-023-36251-3)
Supplement: Supplementary file 1 — Supplementary Information [file 41467_2023_36251_MOESM1_ESM.pdf]

A step-for-step main-group replica of the Fischer carbene synthesis at a borylene carbonyl

Marcel Härterich[1,2], Alexander Matler[1,2], Rian D. Dewhurst[1,2], Andreas Sachs[1,2], Kai Oppel[1,2], Andreas Stoy[1,2], Holger Braunschweig[1,2]*

[1]Institute for Inorganic Chemistry, Julius-Maximilians-Universität Würzburg, Am Hubland, 97074 Würzburg, Germany. [2]Institute for Sustainable Chemistry & Catalysis with Boron, Julius-Maximilians-Universität Würzburg, Am Hubland, 97074 Würzburg, Germany.

Index

NMR spectra of isolated compounds 2

UV-vis spectroscopy 20

IR spectroscopy 22

Computational details 25

Suppplementary methods 35

References 38

NMR spectra of isolated compounds

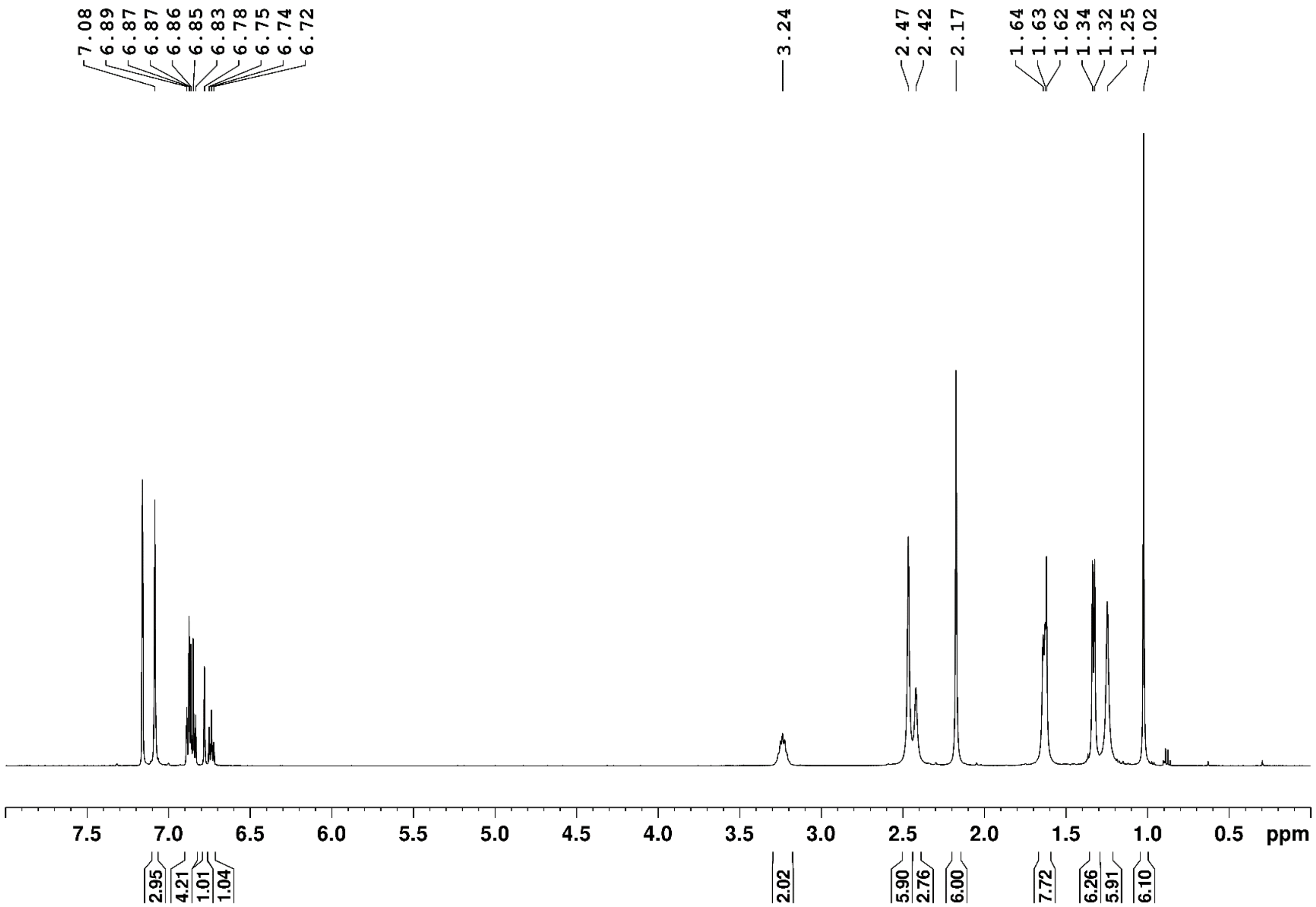

Supplementary Fig. 1. ^{1}H NMR spectrum of (CAAC)B(Dur)=C(Ph)(OMe) (**3a**) in C_6D_6. The additional multiplets at 1.24 and 0.89 ppm belong to residual hexane from washing.

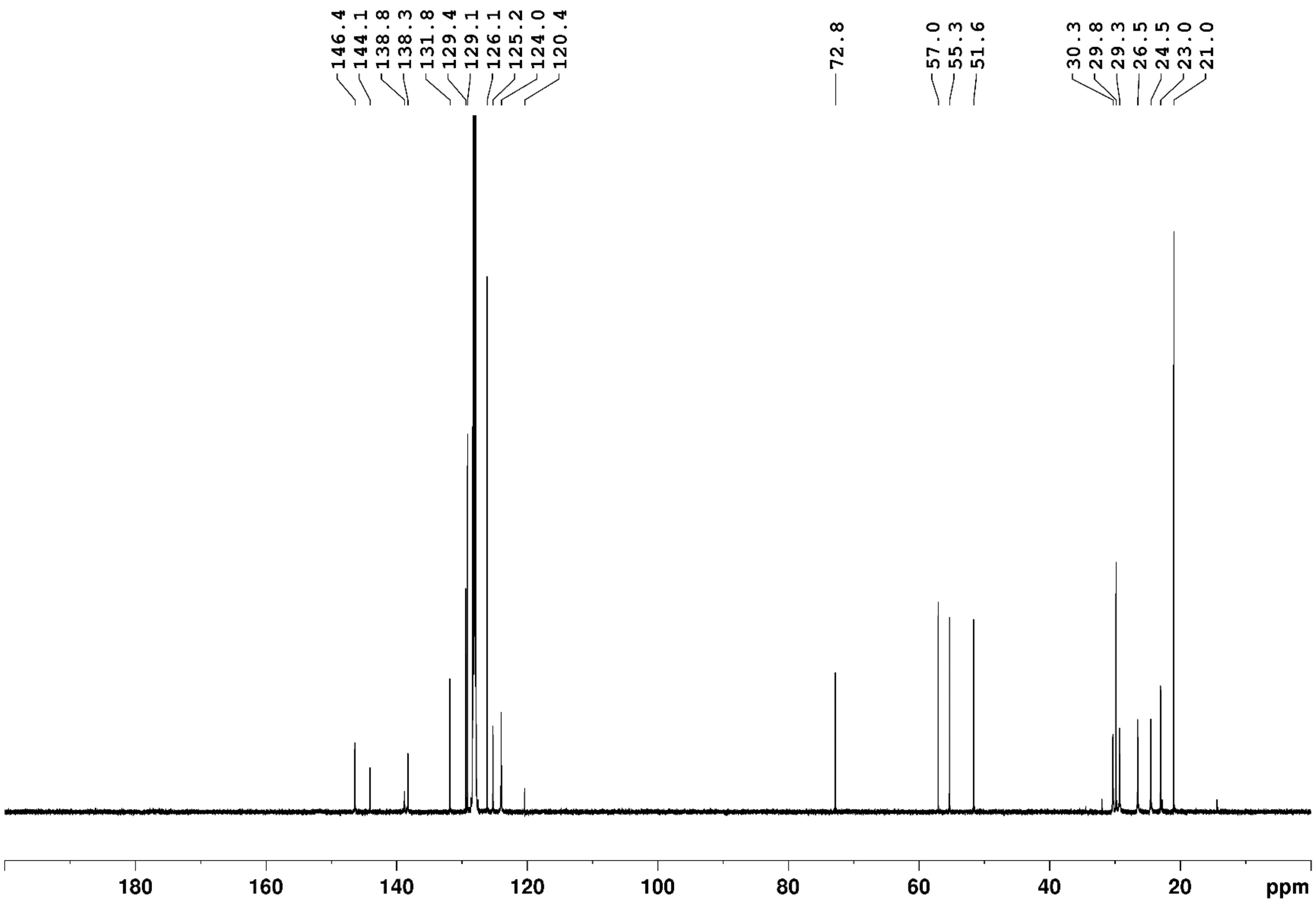

Supplementary Fig. 2. $^{13}C\{^{1}H\}$ NMR spectrum of (CAAC)B(Dur)=C(Ph)(OMe) (**3a**) in C_6D_6. The additional resonances at 32.0, 23.0 and 14.3 ppm belong to residual hexane from washing.

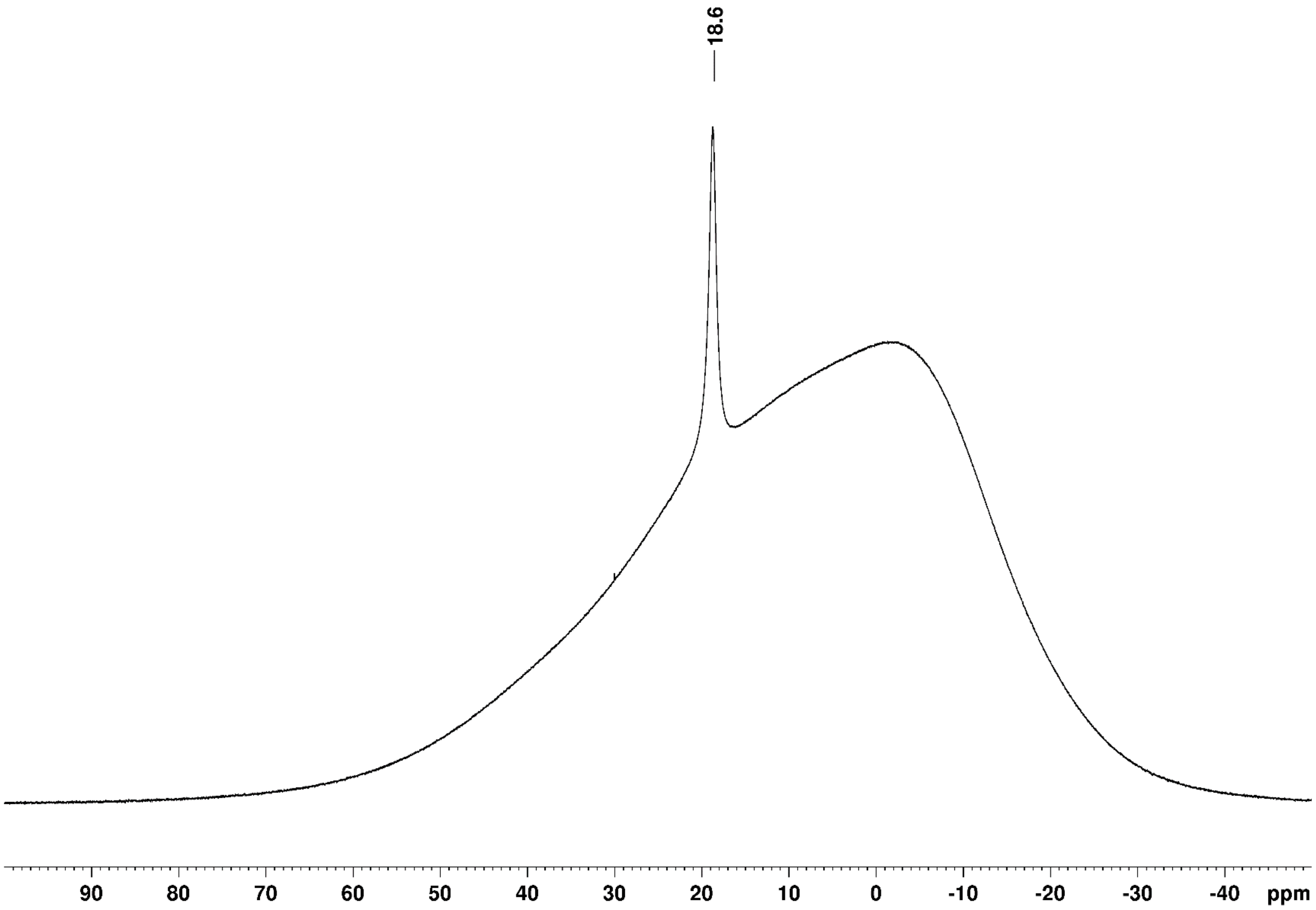

Supplementary Fig. 3. ^{11}B NMR spectrum of (CAAC)B(Dur)=C(Ph)(OMe) (**3a**) in C_6D_6.

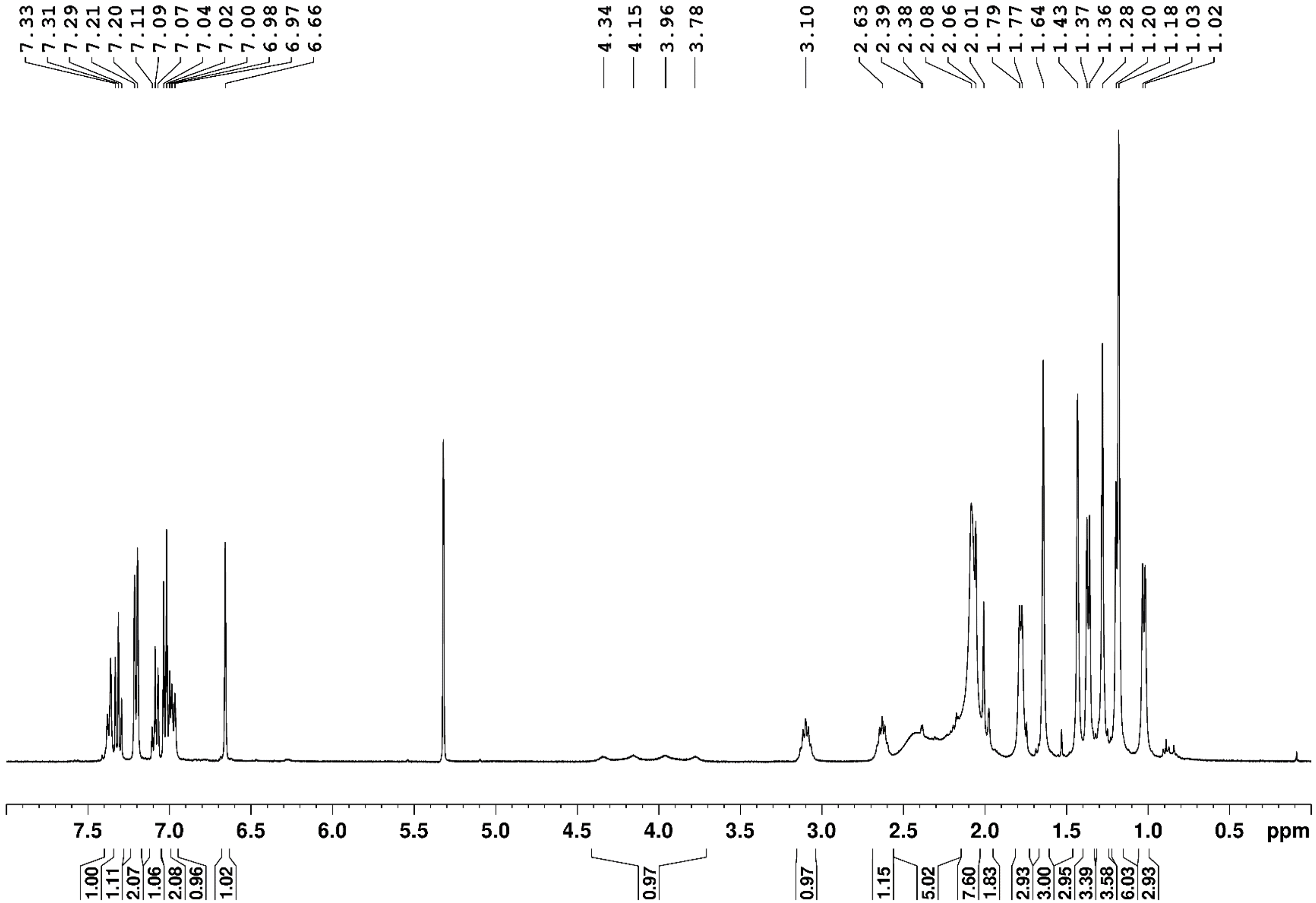

Supplementary Fig. 4. ^{1}H NMR spectrum of (CAAC)B(Dur)(H)C(=O)(Ph) (**4a**) in CD_2Cl_2.

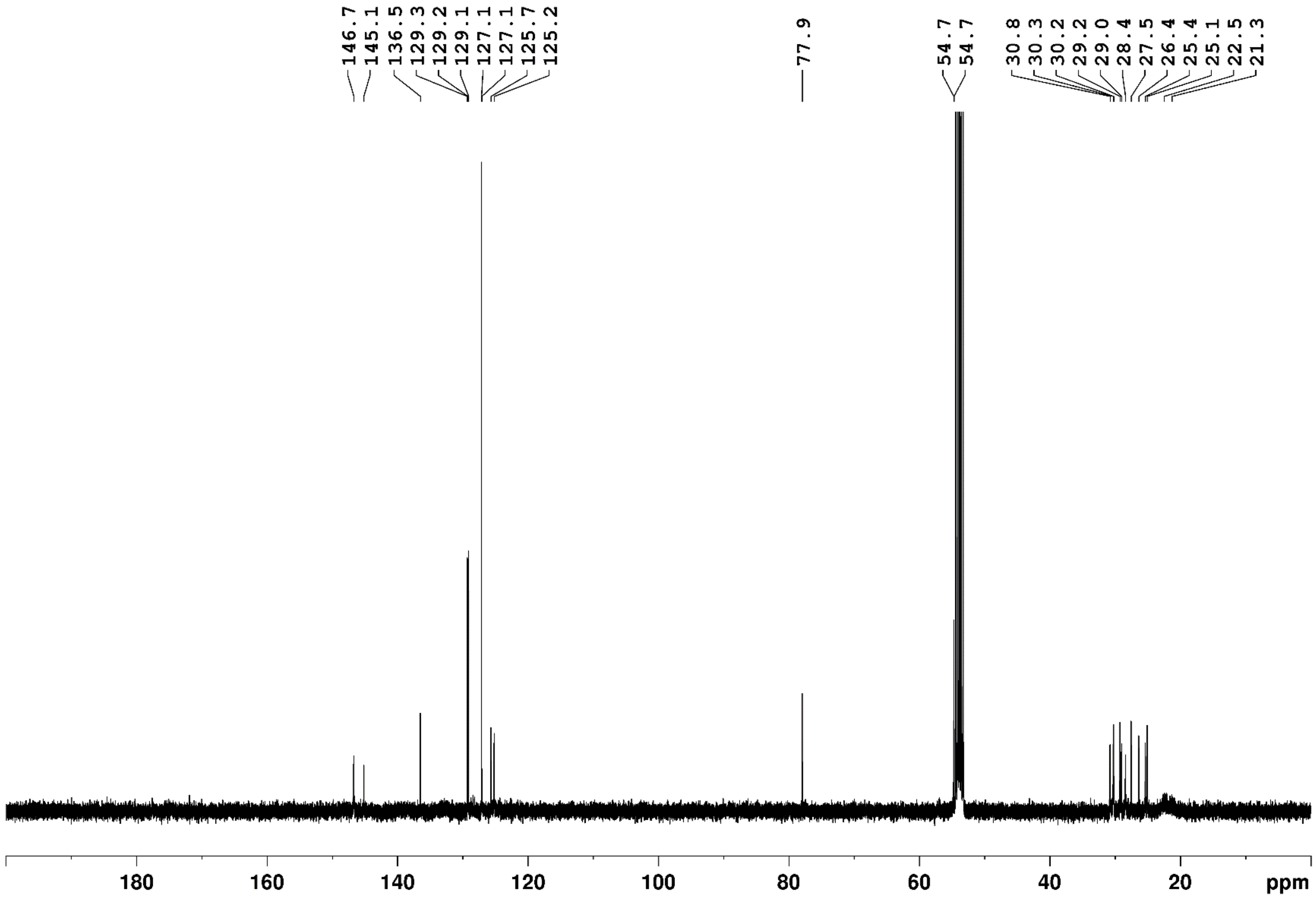

Supplementary Fig. 5. $^{13}C\{^{1}H\}$ NMR spectrum of (CAAC)B(Dur)(H)C(=O)(Ph) (**4a**) in CD_2Cl_2.

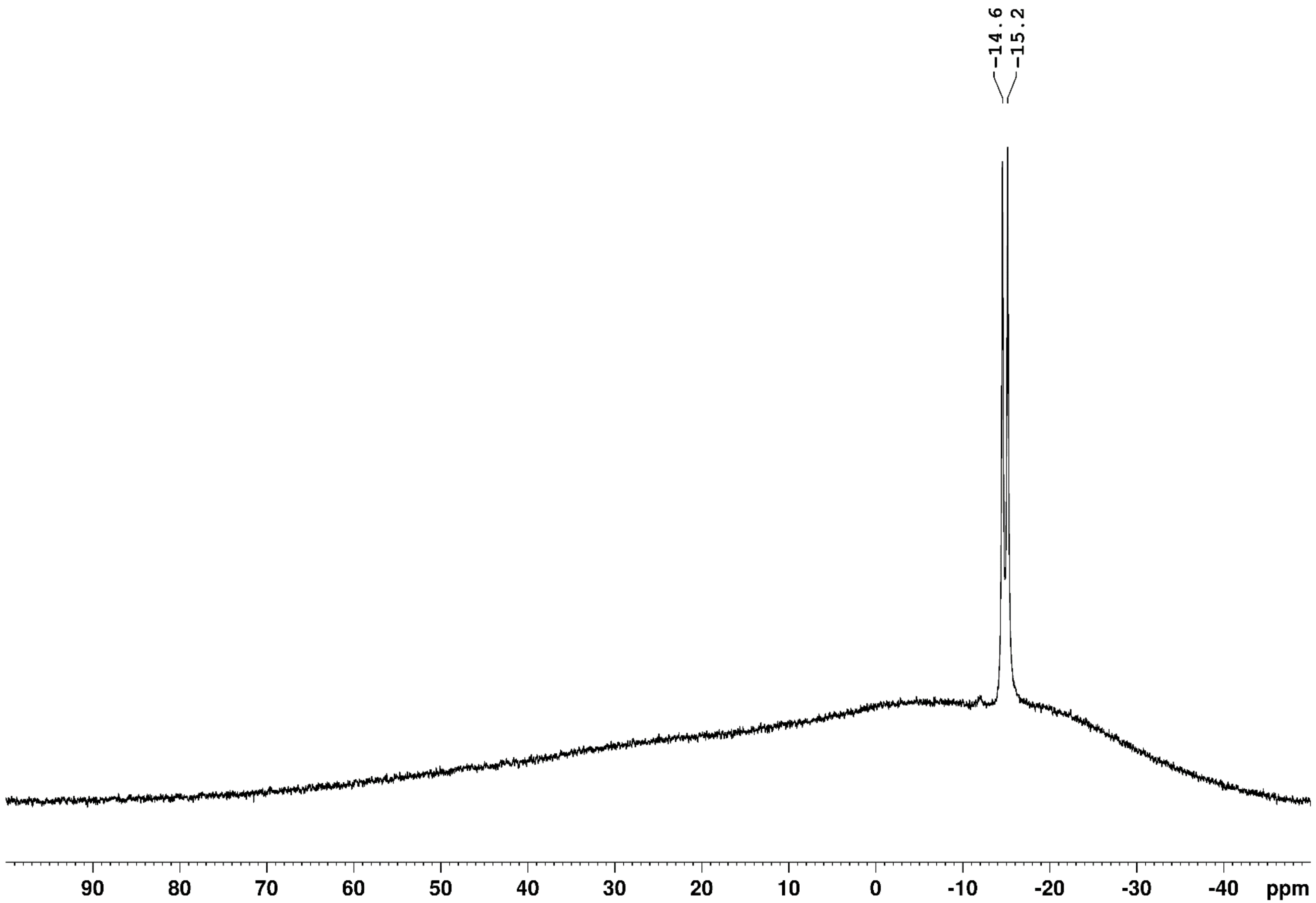

Supplementary Fig. 6. ^{11}B NMR spectrum of (CAAC)B(Dur)(H)C(=O)(Ph) (**4a**) in CD_2Cl_2.

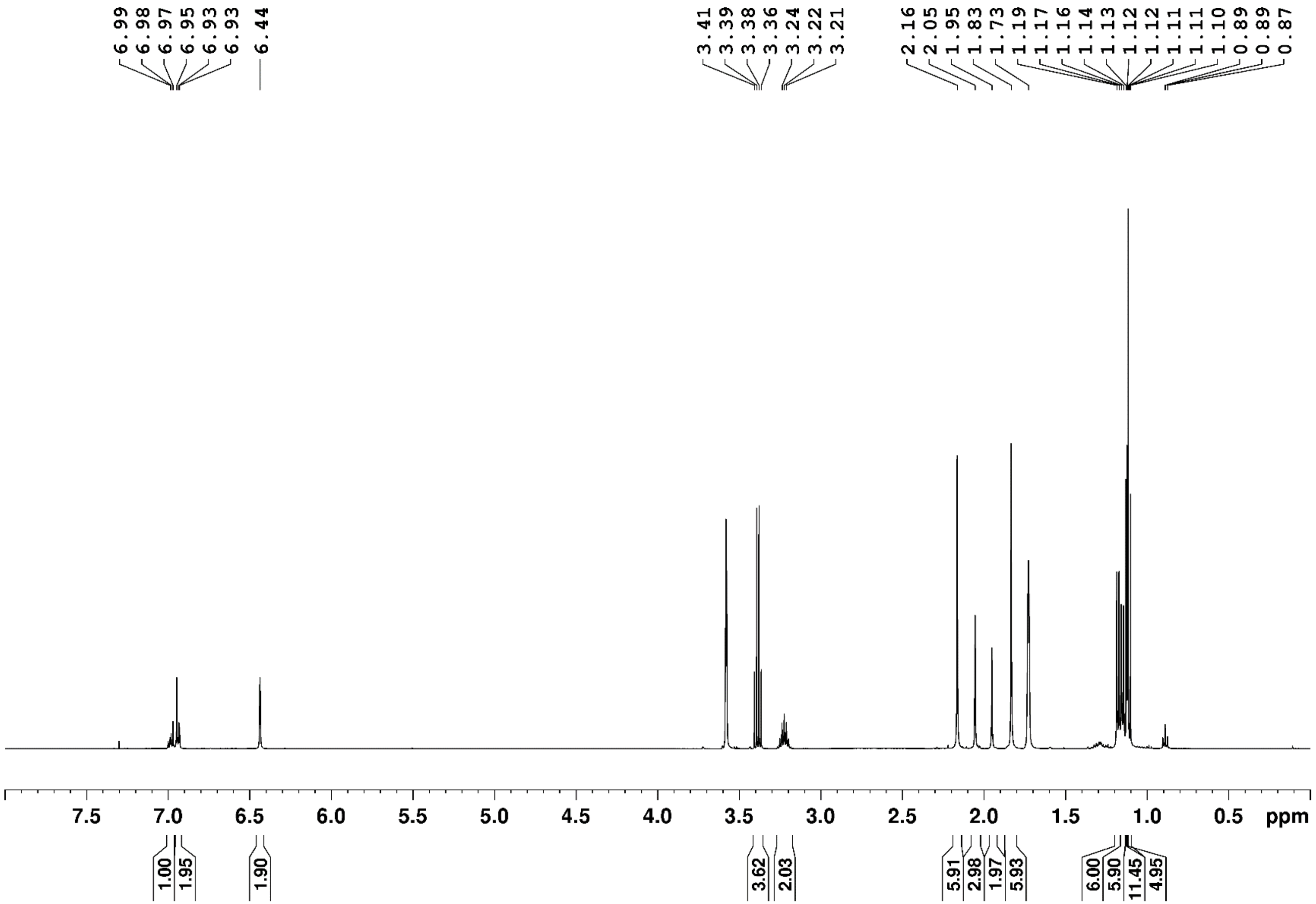

Supplementary Fig. 7. ^{1}H NMR spectrum of (CAAC)B(H)=C(Mes)(OLi) (**2b**) in THF-d_8. The additional multiplets at 1.29 and 0.89 ppm belong to residual hexane from washing.

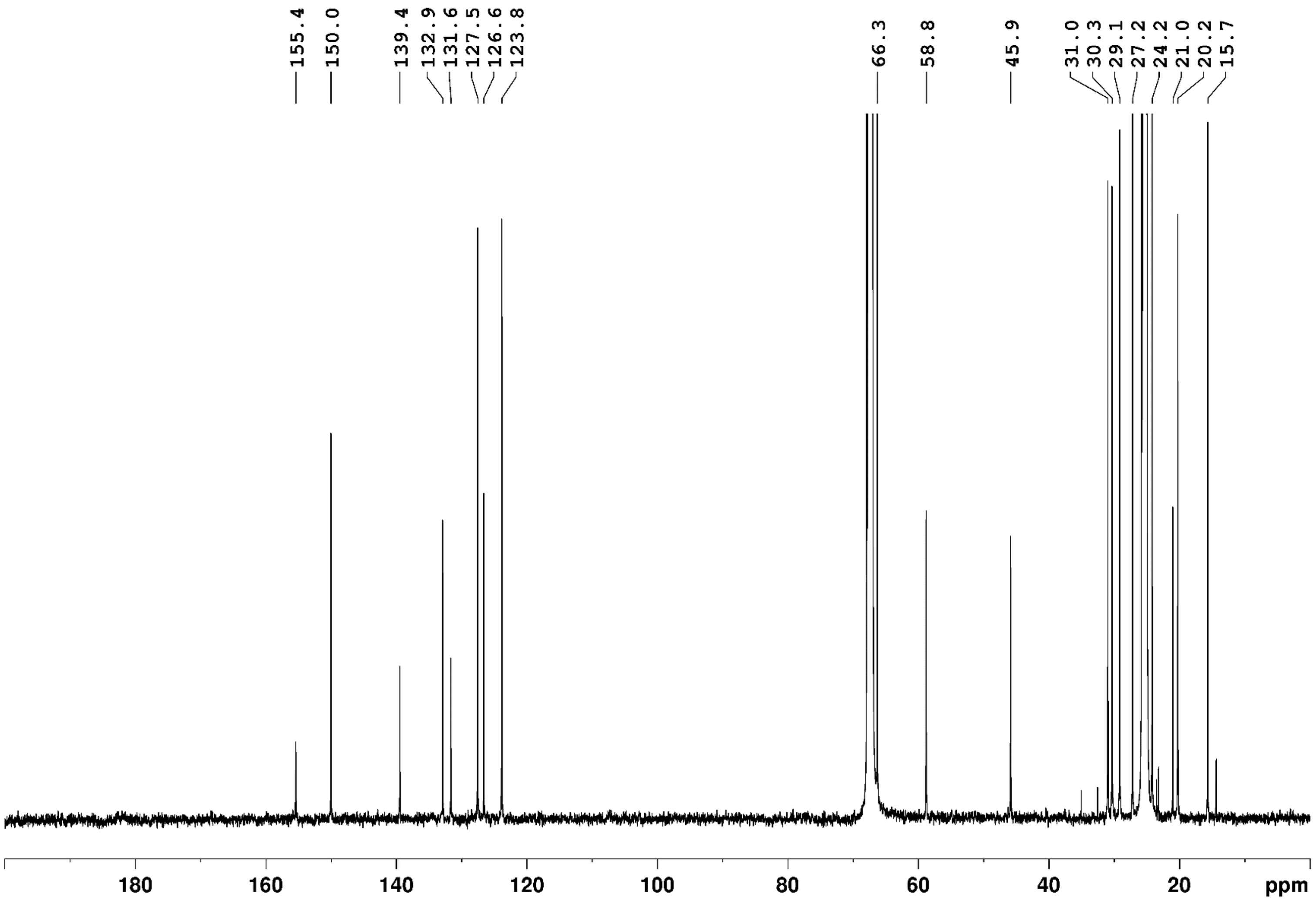

Supplementary Fig. 8. $^{13}C\{^1H\}$ NMR spectrum of (CAAC)B(H)=C(Mes)(OLi) (**2b**) in THF-d_8. The additional resonances at 32.3, 23.3 and 14.2 ppm belong to residual hexane from washing.

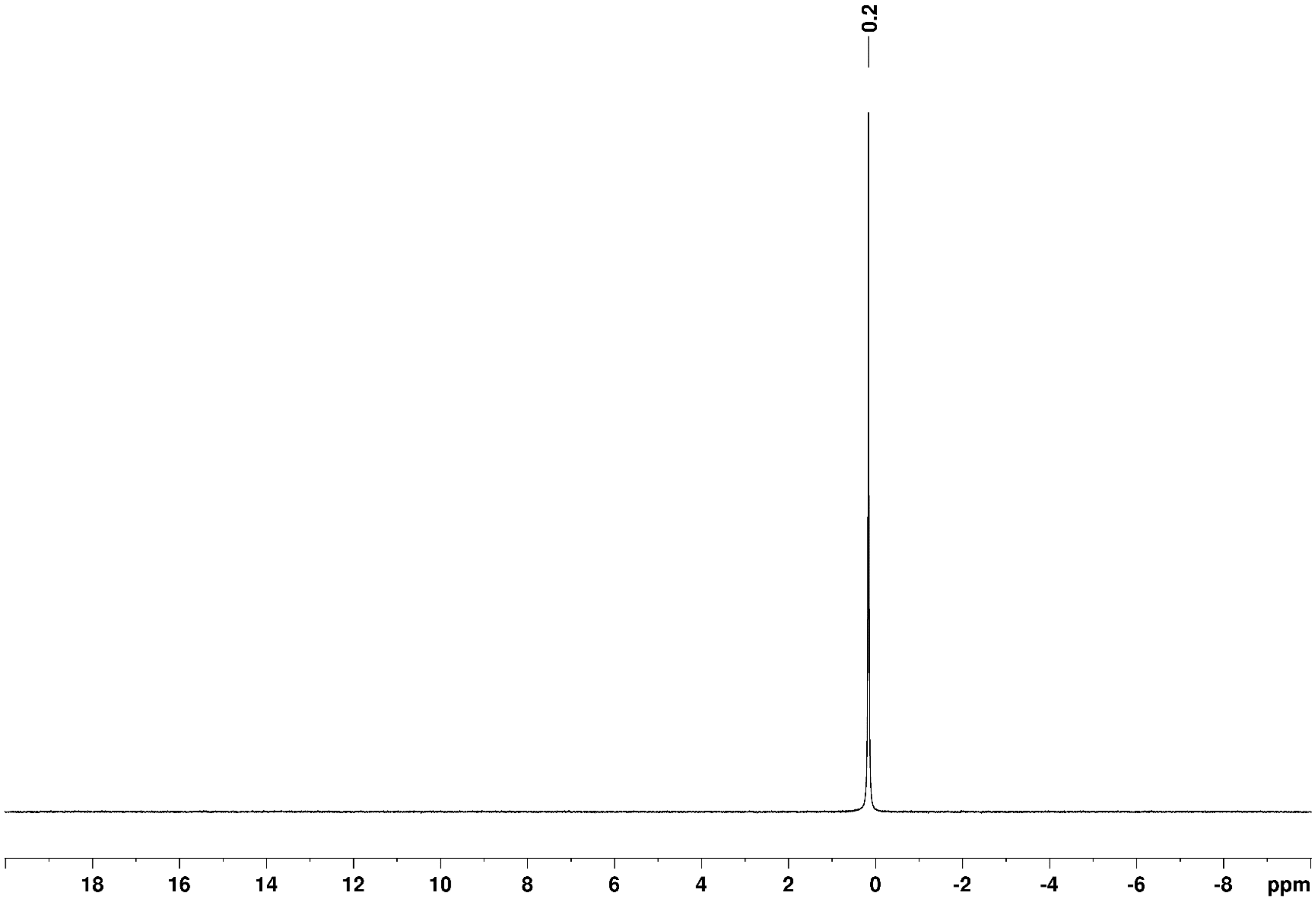

Supplementary Fig. 9. ^{7}Li NMR spectrum of (CAAC)B(H)=C(Mes)(OLi) (**2b**) in THF-d_8.

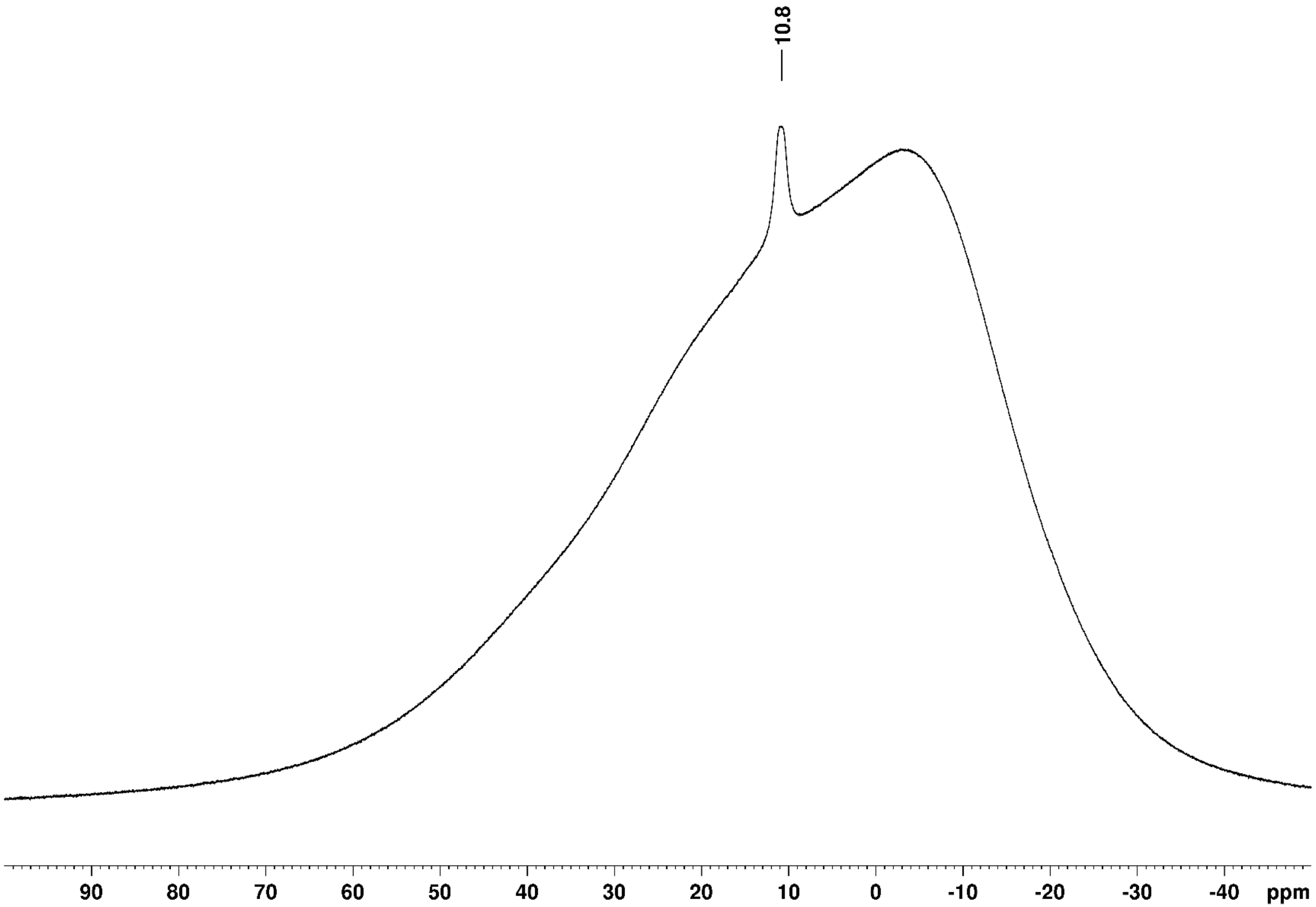

Supplementary Fig. 10. ^{11}B NMR spectrum of (CAAC)B(H)=C(Mes)(OLi) (**2b**) in THF-d_8.

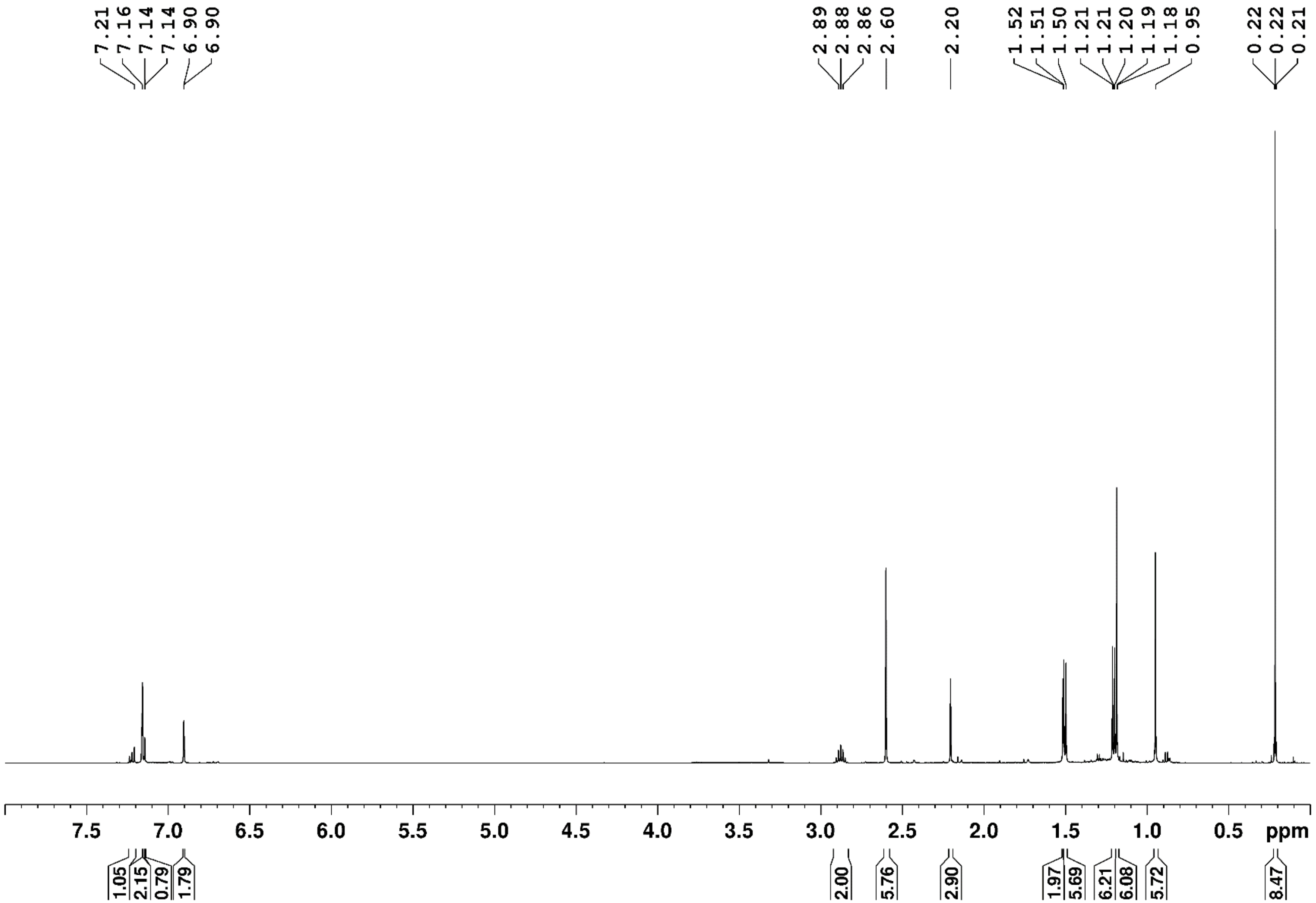

Supplementary Fig. 11. ^{1}H NMR spectrum of (CAAC)B(H)=C(Mes)($OSiMe_3$) (**3b**) in C_6D_6. The additional multiplets at 1.24 and 0.89 ppm belong to residual hexane from washing.

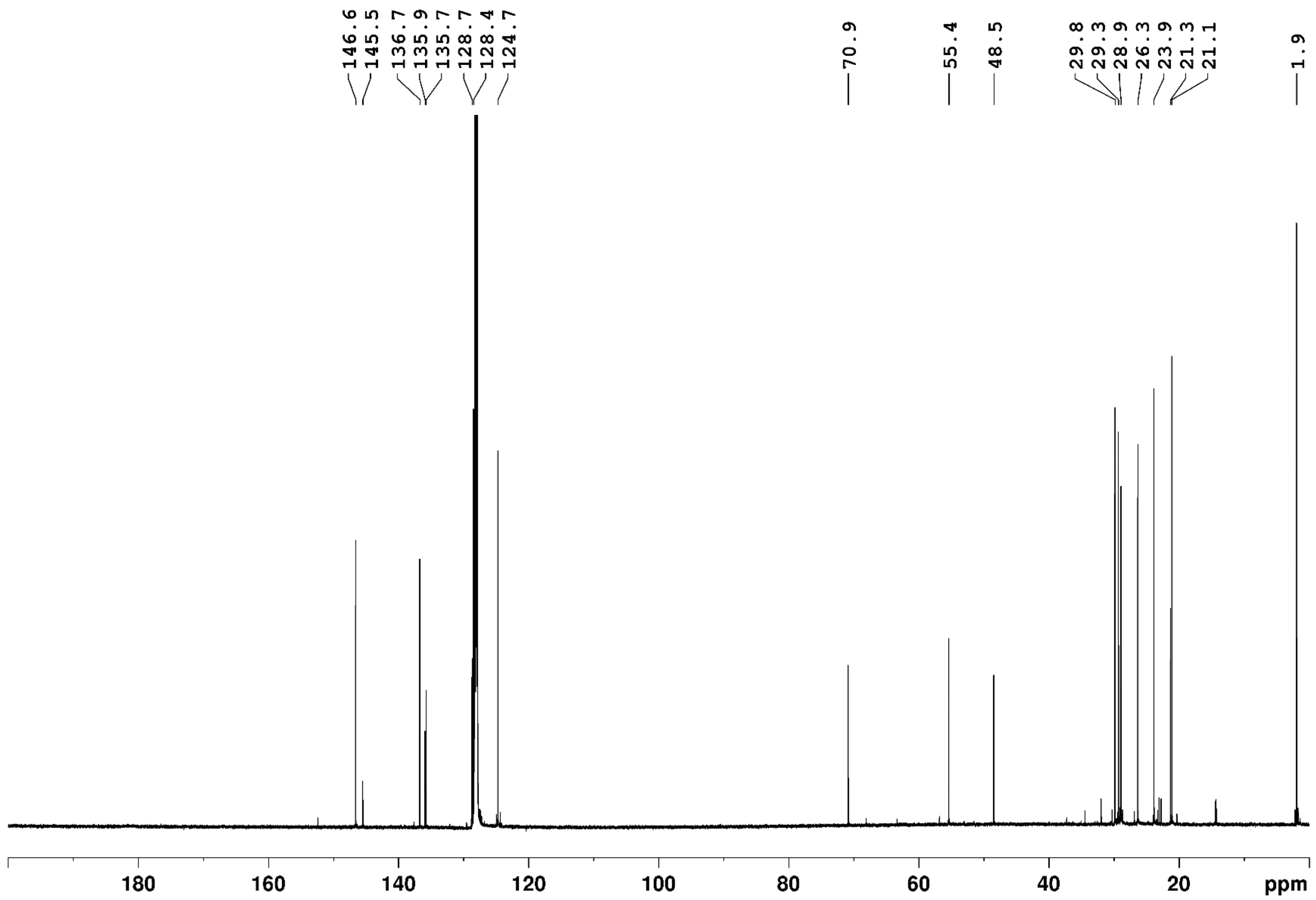

Supplementary Fig. 12. $^{13}C\{^1H\}$ NMR spectrum of (CAAC)B(H)=C(Mes)($OSiMe_3$) (**3b**) in C_6D_6. The additional resonances at 32.0, 23.0 and 14.3 ppm belong to residual hexane from washing.

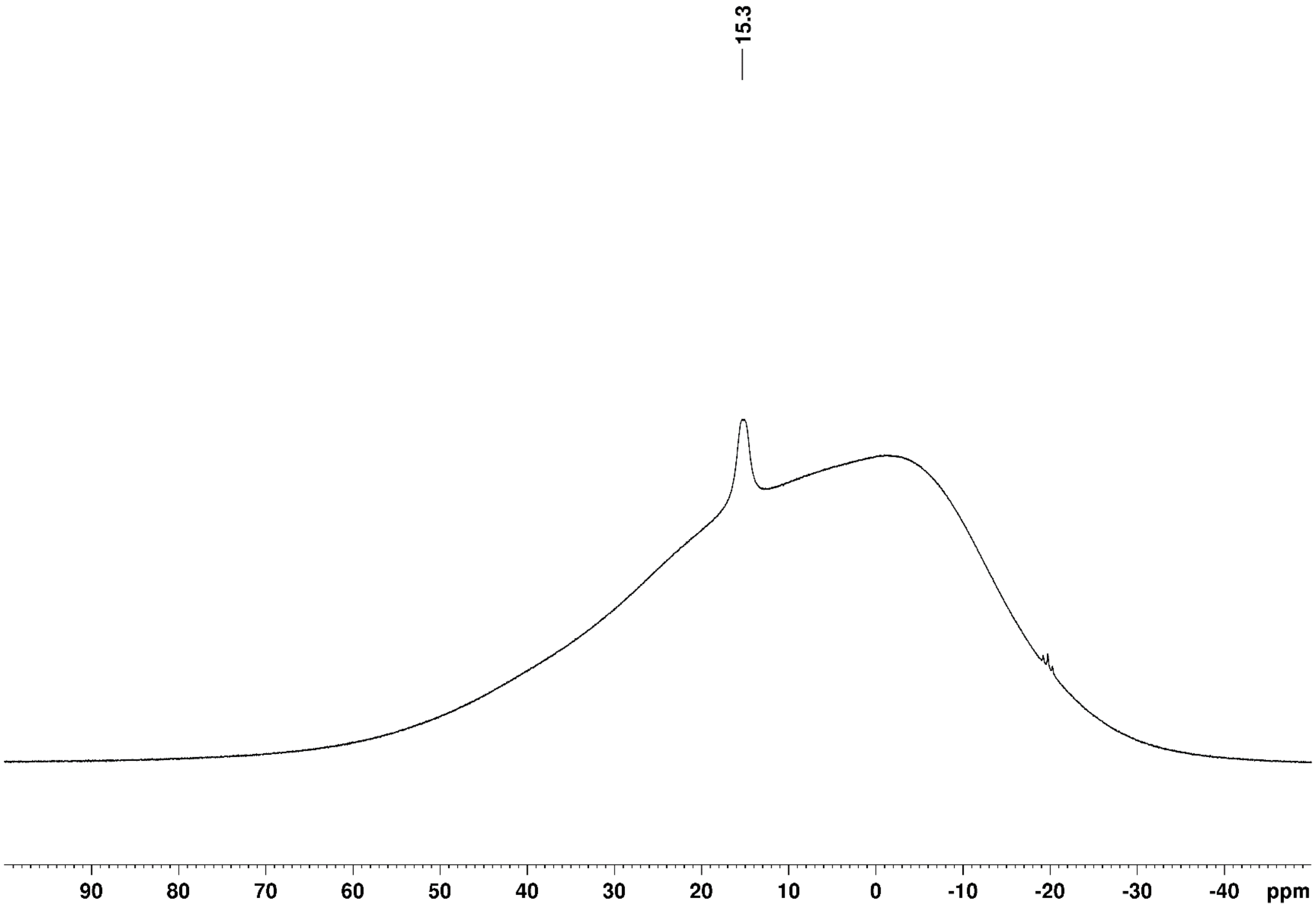

Supplementary Fig. 13. ^{11}B NMR spectrum of (CAAC)B(H)=C(Mes)($OSiMe_3$) (**3b**) in C_6D_6.

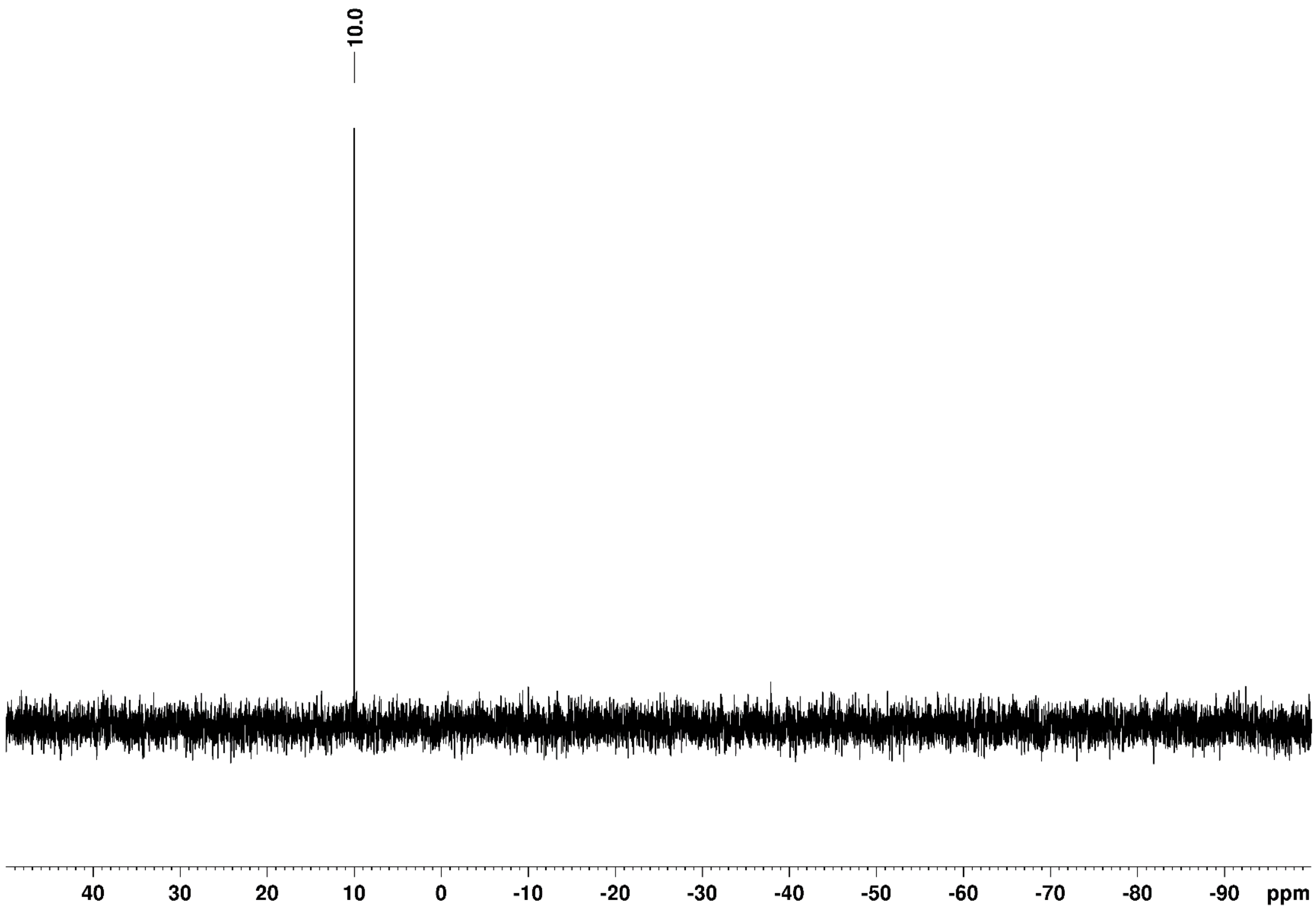

Supplementary Fig. 14. ^{29}Si NMR spectrum of (CAAC)B(H)=C(Mes)($OSiMe_3$) (**3b**) in C_6D_6.

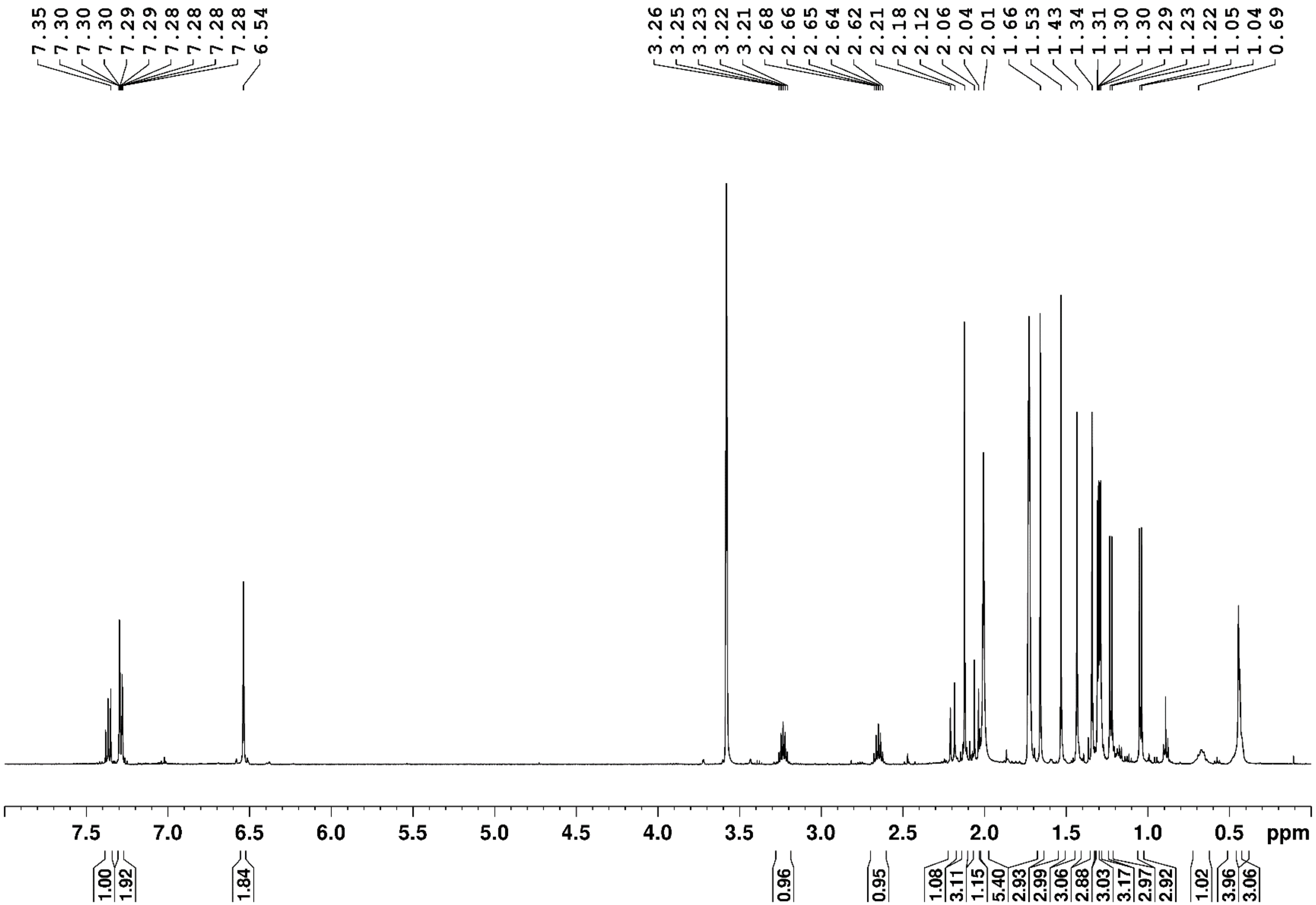

Supplementary Fig. 15. ^{1}H NMR spectrum of (CAAC)B(H)(Et)C(=O)(Mes) (**4b**) in THF-d_8. The additional multiplets at 1.29 and 0.89 ppm belong to residual hexane from washing.

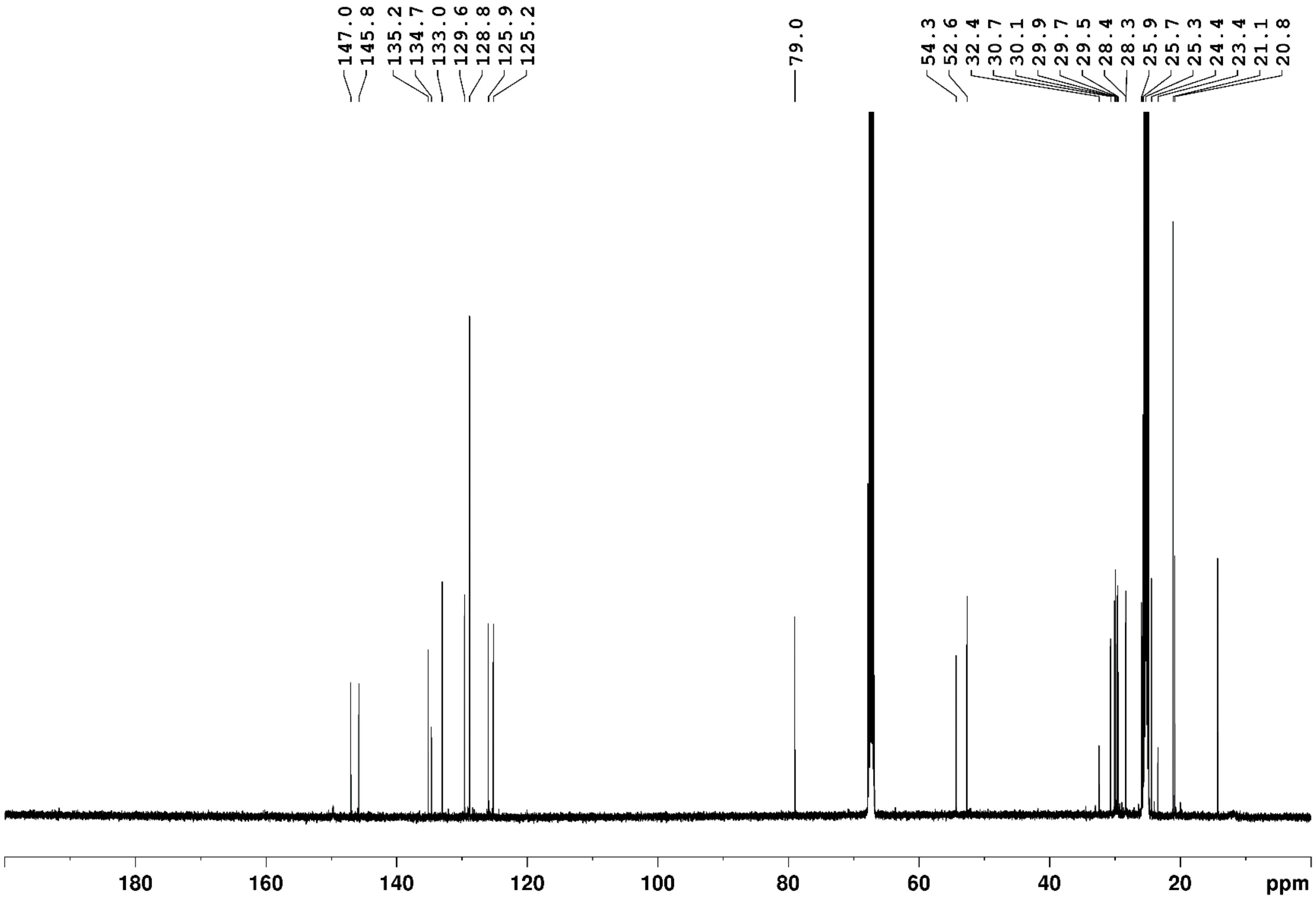

Supplementary Fig. 16. $^{13}C\{^{1}H\}$ NMR spectrum of (CAAC)B(H)(Et)C(=O)(Mes) (**4b**) in THF-d_8. The additional resonances at 32.3, 23.3 and 14.2 ppm belong to residual hexane from washing.

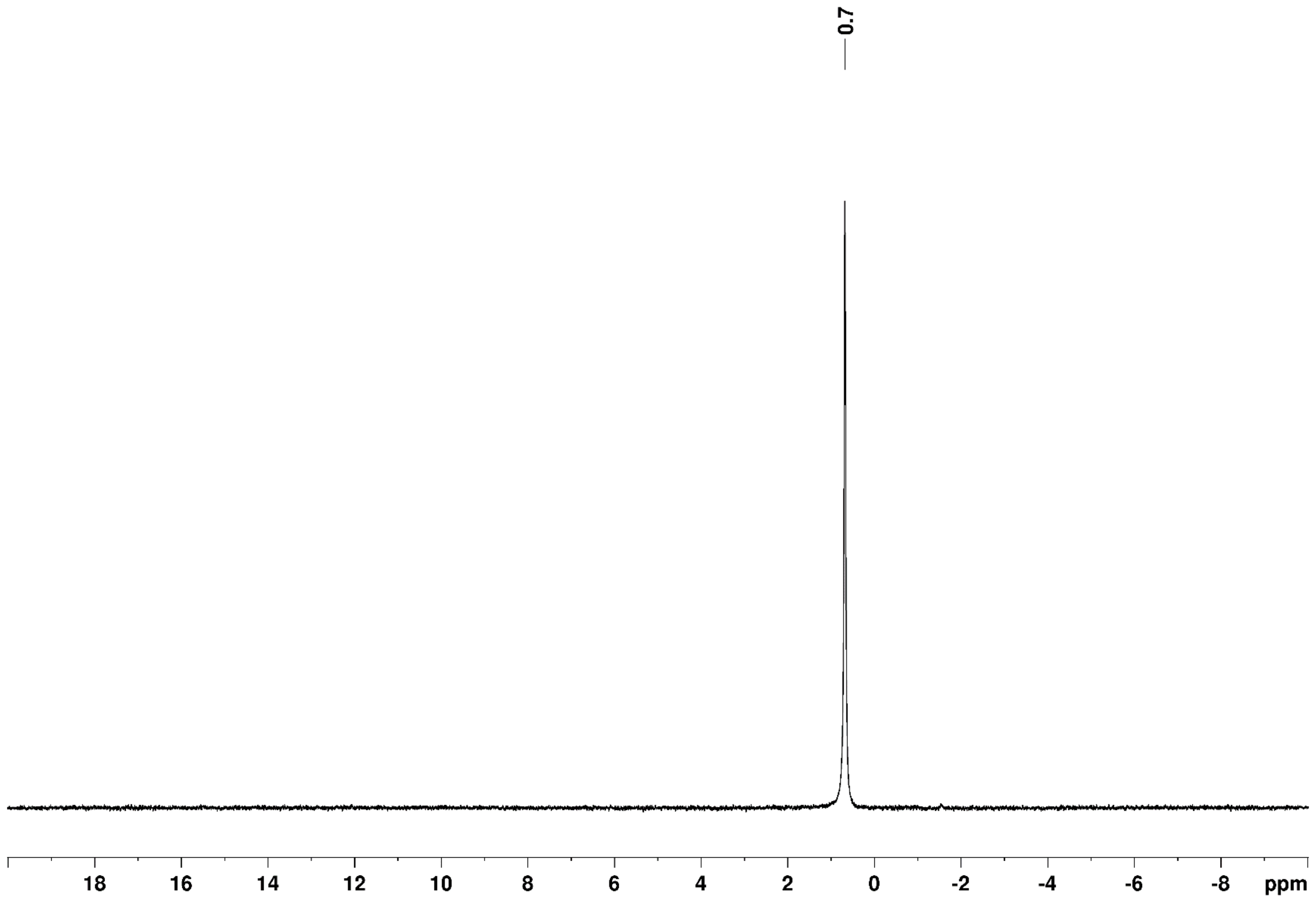

Supplementary Fig. 17. ^{7}Li NMR spectrum of (CAAC)B(H)(Et)C(=O)(Mes) (**4b**) in THF-d_8.

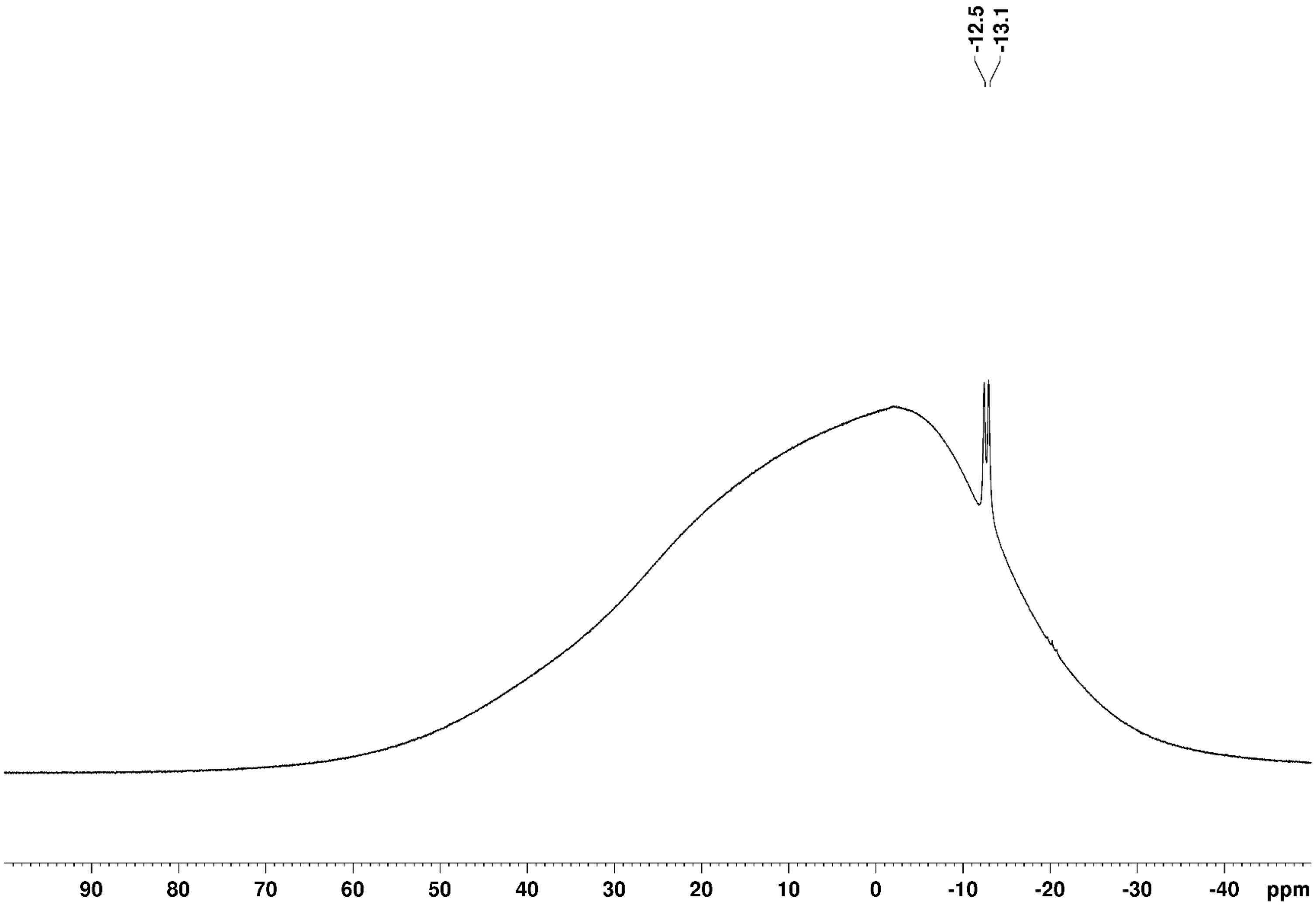

Supplementary Fig. 18. ^{11}B NMR spectrum of (CAAC)B(H)(Et)C(=O)(Mes) (**4b**) in THF-d_8.

UV-vis spectroscopy

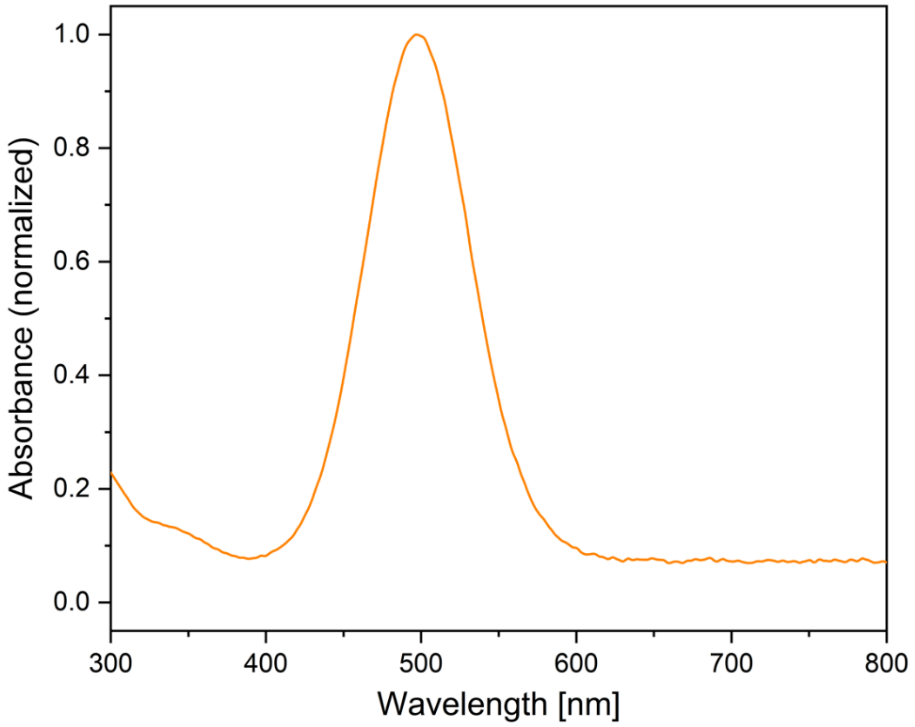

Supplementary Fig. 19. UV-vis absorption spectrum of (CAAC)B(Dur)=C(Ph)(OMe) (**3a**) in THF (λ_{max} = 497 nm).

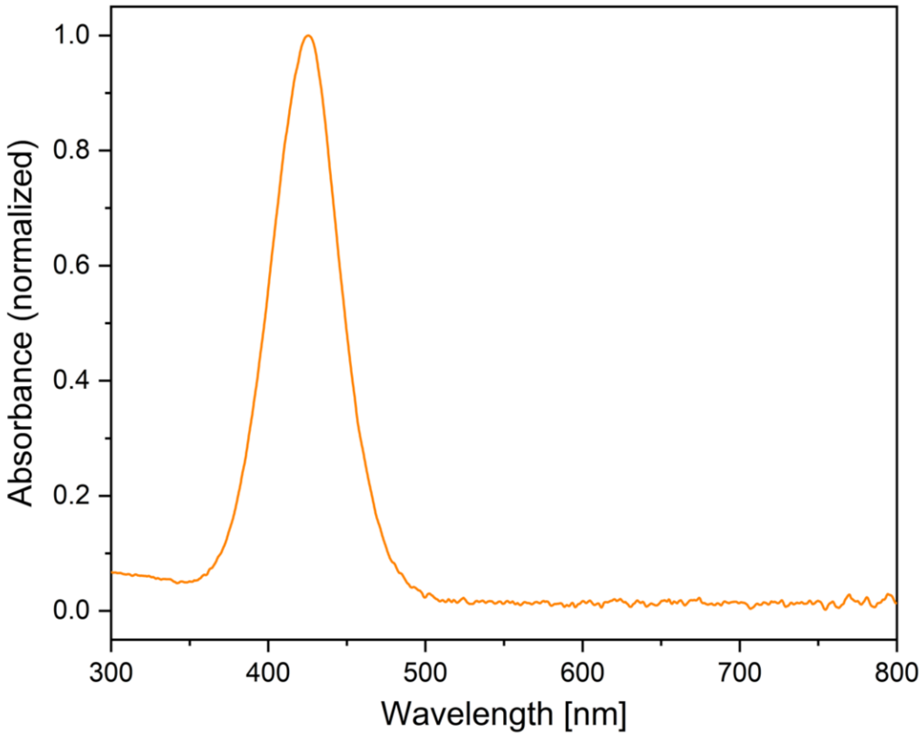

Supplementary Fig. 20. UV-vis absorption spectrum of (CAAC)B(H)=C(Mes)(OLi) (**2b**) in THF (λ_{max} = 425 nm).

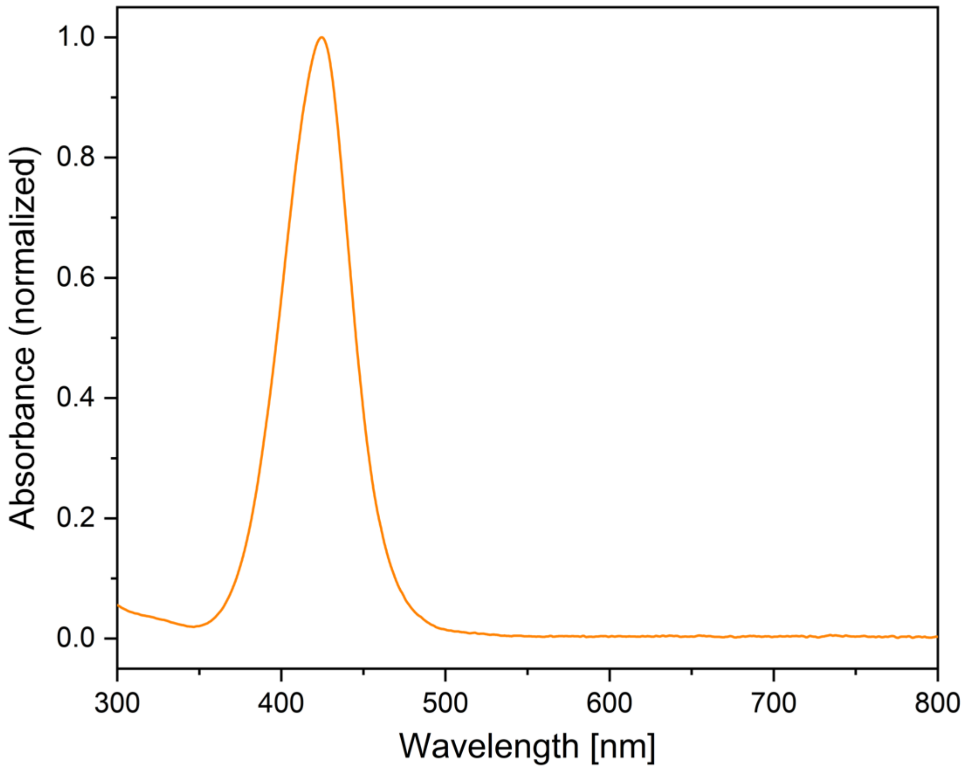

Supplementary Fig. 21. UV-vis absorption spectrum of (CAAC)B(H)=C(Mes)(OSiMe$_3$) (**3b**) in THF (λ_{max} = 426 nm).

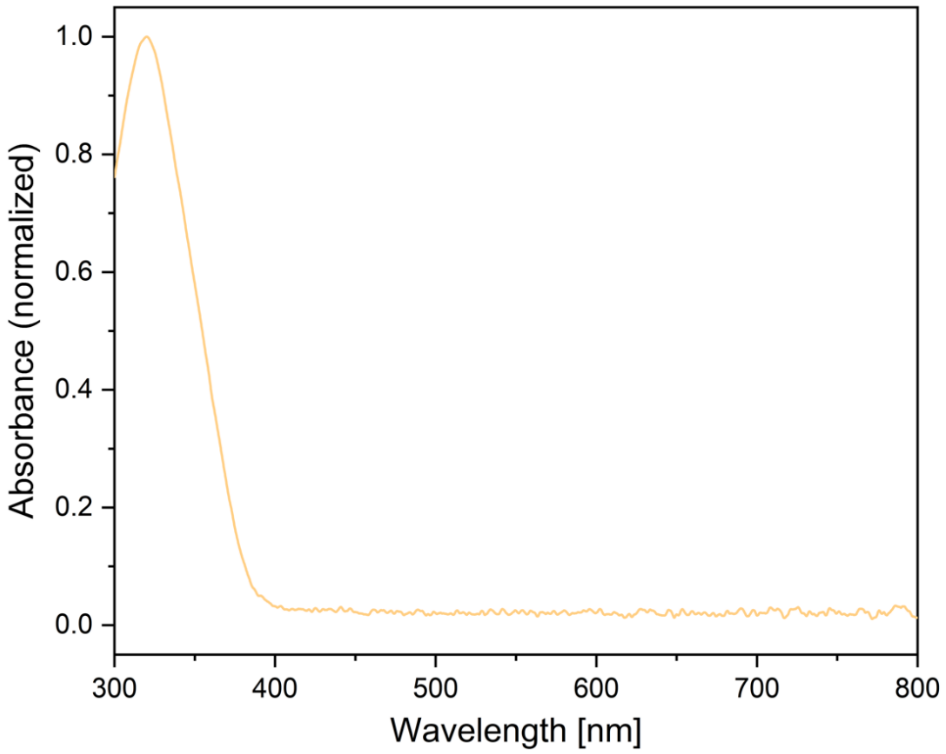

Supplementary Fig. 22. UV-vis absorption spectrum of (CAAC)B(H)(Et)C(=O)(Mes) (**4b**) in THF (λ_{max} = 320 nm).

IR spectroscopy

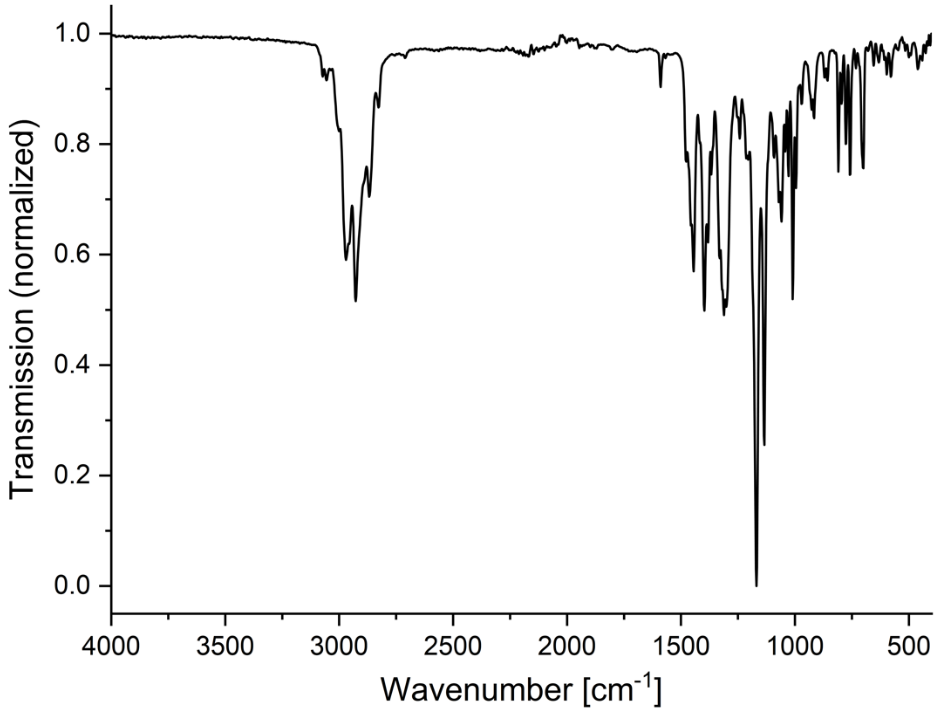

Supplementary Fig. 23. Solid-state IR spectrum of (CAAC)B(Dur)=C(Ph)(OMe) (**3a**).

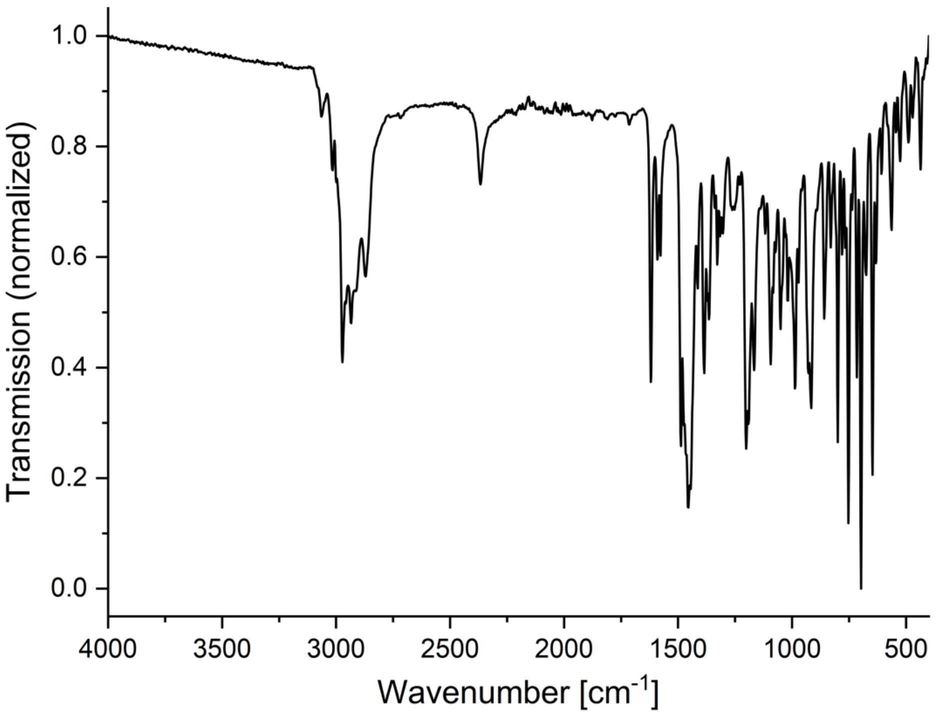

Supplementary Fig. 24. Solid-state IR spectrum of (CAAC)B(Dur)(H)C(=O)(Ph) (**4a**).

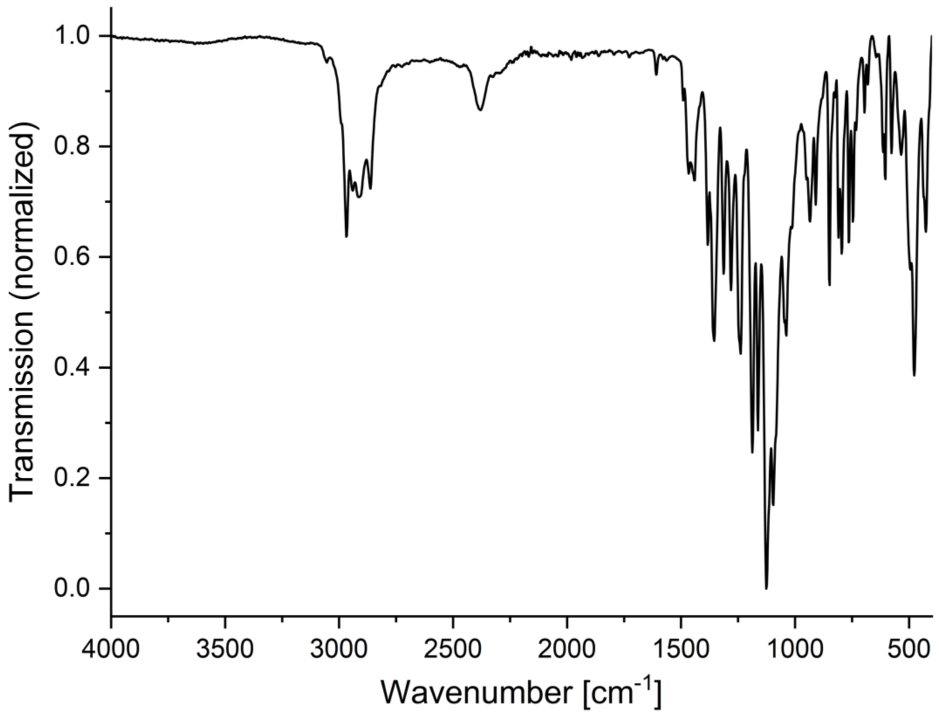

Supplementary Fig. 25. Solid-state IR spectrum of (CAAC)B(H)=C(Mes)(OLi) (**2b**).

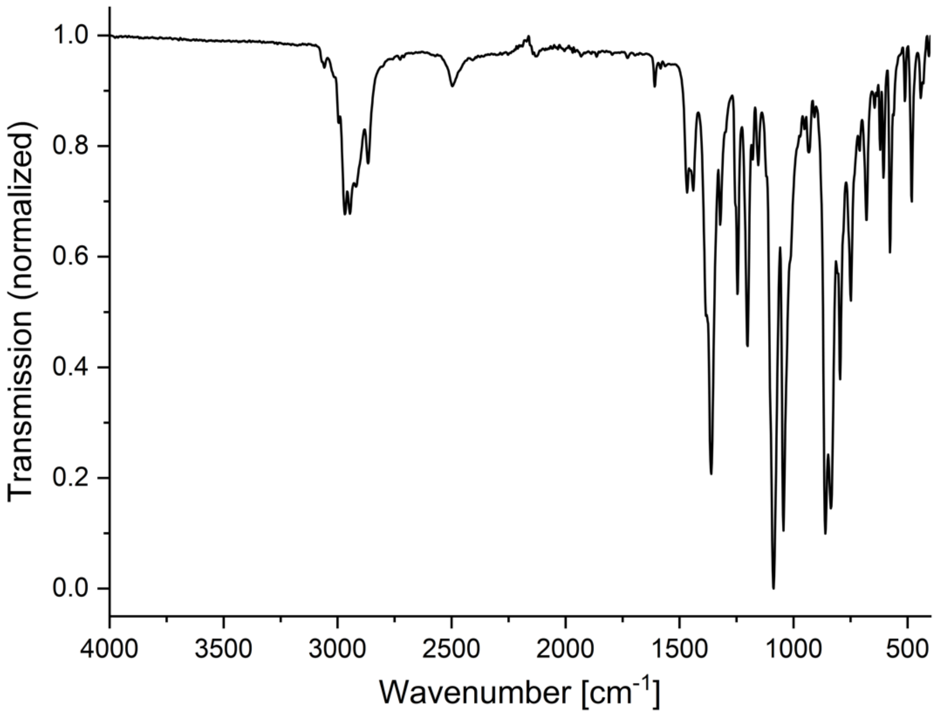

Supplementary Fig. 26. Solid-state IR spectrum of (CAAC)B(H)=C(Mes)($OSiMe_3$) (**3b**).

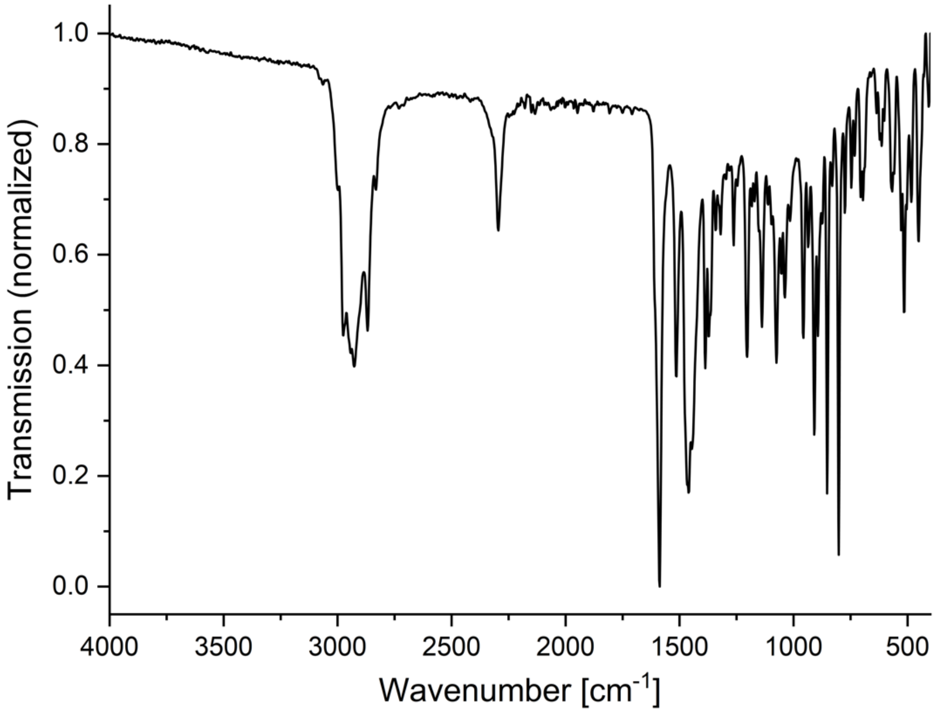

Supplementary Fig. 27. Solid-state IR spectrum of (CAAC)B(H)(Et)C(=O)(Mes) (**4b**).

Computational details

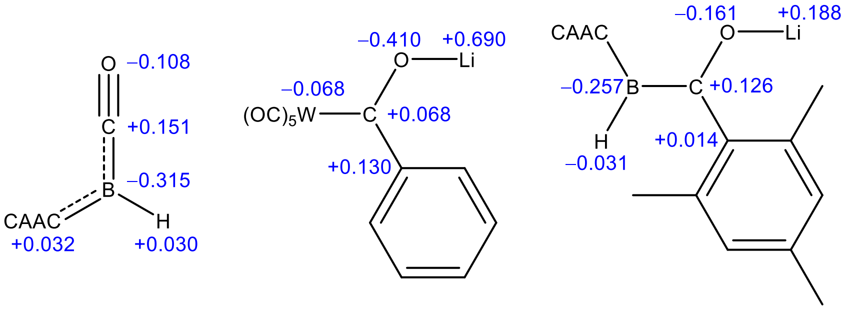

Supplementary Fig. 28. Calculated ADCH charges (level of theory: B3-LYP/def2-SVP with GD3 dispersion correction) for borylene **1b**, an exemplary metal Fischer carbene and a boron analogue.

Cartesian coordinates of optimized structures

Supplementary Table 1. Cartesian coordinates (level of theory: B3-LYP/def2-SVP with GD3 dispersion correction) for borylene **1b**.

C	-2.528815000000	-0.111009000000	-2.534295000000
B	-1.288534000000	-0.098048000000	-1.756873000000
H	-0.286435000000	0.018525000000	-2.417147000000
O	-3.487420000000	-0.103699000000	-3.188526000000
C	-1.338921000000	-0.172399000000	-0.264741000000
N	-0.238312000000	-0.100185000000	0.531089000000
C	-2.578356000000	-0.287010000000	0.630164000000
C	-3.324854000000	1.065474000000	0.659722000000
H	-4.159414000000	1.024369000000	1.379250000000
H	-3.734768000000	1.304878000000	-0.332356000000
H	-2.662341000000	1.893127000000	0.950995000000
C	-3.551302000000	-1.391422000000	0.183348000000
H	-3.028892000000	-2.351966000000	0.057654000000
H	-4.046461000000	-1.149301000000	-0.767306000000
H	-4.340691000000	-1.522442000000	0.942240000000
C	-1.972980000000	-0.641868000000	2.011731000000
H	-2.544193000000	-0.203705000000	2.843494000000
H	-1.994816000000	-1.734677000000	2.143465000000
C	-0.507234000000	-0.166629000000	2.005370000000
C	-0.329972000000	1.205374000000	2.678031000000
H	-0.561719000000	1.121605000000	3.750827000000
H	-0.990276000000	1.969127000000	2.248433000000
H	0.708359000000	1.551044000000	2.582418000000
C	0.416210000000	-1.164398000000	2.716185000000
H	0.178997000000	-1.175934000000	3.791061000000
H	1.473424000000	-0.881341000000	2.603902000000
H	0.286119000000	-2.184080000000	2.333105000000
C	1.088621000000	0.073867000000	0.004420000000
C	1.585794000000	1.376849000000	-0.244432000000
C	2.909628000000	1.506852000000	-0.689504000000
H	3.313663000000	2.503722000000	-0.879856000000
C	3.709967000000	0.390328000000	-0.918317000000
H	4.740078000000	0.514785000000	-1.262227000000
C	3.180752000000	-0.886281000000	-0.742568000000
H	3.796194000000	-1.758503000000	-0.974031000000
C	1.865083000000	-1.072459000000	-0.295810000000
C	0.440783000000	3.199074000000	-1.553162000000
H	-0.035338000000	2.444164000000	-2.193966000000
H	1.372075000000	3.531080000000	-2.040692000000
H	-0.232778000000	4.069406000000	-1.486094000000
C	0.720216000000	2.629850000000	-0.149144000000
H	-0.249940000000	2.338321000000	0.270232000000
C	1.327824000000	3.713012000000	0.757123000000
H	1.564058000000	3.330009000000	1.760937000000
H	0.625990000000	4.555144000000	0.870609000000
H	2.258604000000	4.119878000000	0.330254000000
C	1.298058000000	-2.487820000000	-0.243890000000

H	0.305540000000	-2.435178000000	0.223027000000
C	2.165304000000	-3.450012000000	0.584115000000
H	1.665290000000	-4.427136000000	0.683464000000
H	2.361712000000	-3.061206000000	1.594456000000
H	3.139665000000	-3.631470000000	0.102739000000
C	1.088112000000	-3.033335000000	-1.669015000000
H	2.047074000000	-3.115960000000	-2.206681000000
H	0.424390000000	-2.374140000000	-2.246094000000
H	0.633542000000	-4.037236000000	-1.633135000000

Supplementary Table 2. Cartesian coordinates (level of theory: B3-LYP/def2-SVP with GD3 dispersion correction) for **2b**.

B	3.330136000000	-0.627859000000	-0.405446000000
H	3.136364000000	0.528117000000	-0.067491000000
C	2.035898000000	-1.390926000000	-0.562777000000
O	0.892922000000	-0.761872000000	-0.410158000000
Li	0.756844000000	1.090987000000	-0.329834000000
O	1.361266000000	2.611720000000	-1.330937000000
C	1.209873000000	2.800276000000	-2.733187000000
H	2.073432000000	3.374692000000	-3.115898000000
H	0.300979000000	3.398552000000	-2.926488000000
C	1.562080000000	3.805217000000	-0.570450000000
H	0.669084000000	4.447476000000	-0.656206000000
H	2.428445000000	4.353149000000	-0.985565000000
C	1.137823000000	1.441568000000	-3.403905000000
H	2.045123000000	0.856258000000	-3.192808000000
H	1.034850000000	1.559420000000	-4.493568000000
H	0.274524000000	0.862379000000	-3.042405000000
C	1.818141000000	3.412279000000	0.871347000000
H	0.956798000000	2.856125000000	1.273106000000
H	1.949124000000	4.310864000000	1.492274000000
H	2.726362000000	2.798713000000	0.959488000000
B	-3.353848000000	0.727091000000	0.390139000000
H	-3.058336000000	-0.457466000000	0.430067000000
C	-2.126303000000	1.608010000000	0.348993000000
O	-0.937139000000	1.062143000000	0.427763000000
Li	-0.735794000000	-0.804507000000	0.481440000000
O	-1.014201000000	-1.996293000000	1.986237000000
C	-0.798322000000	-1.679316000000	3.358293000000
H	0.092192000000	-2.226115000000	3.724698000000
H	-1.665562000000	-2.026950000000	3.948993000000
C	-1.344658000000	-3.361355000000	1.723373000000
H	-2.210330000000	-3.646672000000	2.347996000000
H	-0.491929000000	-4.006177000000	2.003814000000
C	-0.619434000000	-0.180680000000	3.504865000000
H	0.262207000000	0.169958000000	2.948619000000
H	-0.479751000000	0.077344000000	4.566078000000
H	-1.500029000000	0.353970000000	3.119608000000
C	-1.675914000000	-3.504322000000	0.252779000000
H	-2.528035000000	-2.863386000000	-0.014578000000
H	-1.955520000000	-4.542646000000	0.025824000000
H	-0.807038000000	-3.253263000000	-0.372516000000
N	5.769990000000	-0.241148000000	0.053887000000
C	4.768215000000	-1.059463000000	-0.437615000000
C	5.453187000000	-2.315554000000	-1.013281000000
C	6.951884000000	-1.920066000000	-1.032728000000
H	7.619169000000	-2.781306000000	-0.878448000000
H	7.199462000000	-1.486802000000	-2.015000000000
C	7.140741000000	-0.837573000000	0.038587000000
C	5.241743000000	-3.578301000000	-0.151547000000
H	4.204498000000	-3.923614000000	-0.205805000000

H	5.491686000000	-3.410434000000	0.905062000000
H	5.885030000000	-4.393161000000	-0.523652000000
C	4.970926000000	-2.615290000000	-2.443105000000
H	5.582140000000	-3.418406000000	-2.890094000000
H	5.050285000000	-1.721666000000	-3.081170000000
H	3.925264000000	-2.945674000000	-2.445011000000
C	7.507827000000	-1.410239000000	1.421919000000
H	7.590552000000	-0.597769000000	2.157740000000
H	8.480283000000	-1.924553000000	1.373380000000
H	6.761042000000	-2.125598000000	1.786514000000
C	8.242454000000	0.155737000000	-0.354404000000
H	8.106548000000	0.539429000000	-1.372416000000
H	9.215633000000	-0.358032000000	-0.319977000000
H	8.285713000000	1.009121000000	0.338520000000
N	-5.728340000000	-0.002006000000	0.025416000000
C	-4.819808000000	1.017366000000	0.246947000000
C	-5.631583000000	2.325346000000	0.352033000000
C	-7.092555000000	1.823812000000	0.476394000000
H	-7.350439000000	1.723877000000	1.542826000000
H	-7.817802000000	2.520432000000	0.029649000000
C	-7.144748000000	0.433718000000	-0.170106000000
C	-5.257867000000	3.139591000000	1.601504000000
H	-4.255627000000	3.571911000000	1.506397000000
H	-5.279665000000	2.509992000000	2.504251000000
H	-5.971622000000	3.970182000000	1.738915000000
C	-5.475074000000	3.232000000000	-0.887094000000
H	-6.198146000000	4.063082000000	-0.834161000000
H	-5.654751000000	2.690474000000	-1.826139000000
H	-4.471698000000	3.667947000000	-0.930917000000
C	-7.511690000000	0.475263000000	-1.666428000000
H	-6.821043000000	1.099677000000	-2.245797000000
H	-8.528365000000	0.878199000000	-1.795197000000
H	-7.493382000000	-0.538948000000	-2.089761000000
C	-8.166821000000	-0.471535000000	0.530949000000
H	-8.121427000000	-1.504239000000	0.154706000000
H	-9.179280000000	-0.084396000000	0.337516000000
H	-8.020416000000	-0.489533000000	1.617476000000
C	5.536339000000	1.026177000000	0.682164000000
C	5.131936000000	1.104347000000	2.038370000000
C	5.045674000000	2.370960000000	2.636317000000
H	4.746996000000	2.446934000000	3.684510000000
C	5.311877000000	3.534353000000	1.917807000000
H	5.244064000000	4.509801000000	2.406642000000
C	5.621528000000	3.448906000000	0.561952000000
H	5.772336000000	4.365300000000	-0.013293000000
C	5.724174000000	2.206784000000	-0.079774000000
C	4.695331000000	-0.114283000000	2.846318000000
H	4.879128000000	-1.005531000000	2.233699000000
C	5.920887000000	2.162185000000	-1.591196000000
H	6.140553000000	1.122245000000	-1.864983000000
C	3.177954000000	-0.061018000000	3.110913000000
H	2.909033000000	0.817023000000	3.720812000000
H	2.849858000000	-0.961164000000	3.655415000000
H	2.623663000000	-0.015371000000	2.163700000000
C	5.464489000000	-0.269767000000	4.168068000000

H	6.552940000000	-0.291102000000	4.011738000000
H	5.175114000000	-1.207109000000	4.670860000000
H	5.244489000000	0.555743000000	4.864305000000
C	4.603158000000	2.530458000000	-2.292212000000
H	4.344262000000	3.585309000000	-2.101928000000
H	3.781802000000	1.903475000000	-1.922046000000
H	4.689382000000	2.396418000000	-3.383544000000
C	7.071859000000	3.048629000000	-2.089468000000
H	7.237202000000	2.890517000000	-3.167707000000
H	8.014129000000	2.834754000000	-1.563253000000
H	6.849045000000	4.119012000000	-1.950517000000
C	-5.377701000000	-1.381680000000	-0.139970000000
C	-4.958630000000	-1.878810000000	-1.399220000000
C	-4.809244000000	-3.266040000000	-1.550176000000
H	-4.502155000000	-3.668107000000	-2.518424000000
C	-5.016791000000	-4.138709000000	-0.484237000000
H	-4.901052000000	-5.216334000000	-0.626765000000
C	-5.319979000000	-3.626787000000	0.776156000000
H	-5.417395000000	-4.309123000000	1.623673000000
C	-5.490931000000	-2.249666000000	0.975147000000
C	-4.566904000000	-0.966047000000	-2.558243000000
H	-4.830292000000	0.061798000000	-2.278337000000
C	-5.696142000000	-1.711619000000	2.387750000000
H	-6.000538000000	-0.660638000000	2.297959000000
C	-5.285795000000	-1.308167000000	-3.872845000000
H	-4.981383000000	-2.295392000000	-4.256472000000
H	-5.036048000000	-0.566362000000	-4.648887000000
H	-6.379193000000	-1.319901000000	-3.754360000000
C	-3.038238000000	-0.993764000000	-2.760800000000
H	-2.522804000000	-0.676266000000	-1.844559000000
H	-2.744533000000	-0.306615000000	-3.571002000000
H	-2.687410000000	-2.003598000000	-3.029712000000
C	-4.359411000000	-1.716901000000	3.150291000000
H	-4.019509000000	-2.750711000000	3.328059000000
H	-4.470081000000	-1.227129000000	4.132182000000
H	-3.584116000000	-1.187510000000	2.579983000000
C	-6.776000000000	-2.462103000000	3.181517000000
H	-7.728106000000	-2.515461000000	2.632639000000
H	-6.962206000000	-1.958806000000	4.144229000000
H	-6.466808000000	-3.494629000000	3.411685000000
C	1.811104000000	-2.862289000000	-0.776026000000
C	1.372112000000	-3.325152000000	-2.035861000000
C	1.070541000000	-4.683353000000	-2.200520000000
H	0.741037000000	-5.037871000000	-3.182559000000
C	1.161296000000	-5.593923000000	-1.139692000000
C	1.553146000000	-5.105441000000	0.113006000000
H	1.603138000000	-5.794314000000	0.962547000000
C	1.873668000000	-3.755696000000	0.314923000000
C	1.182068000000	-2.352424000000	-3.174199000000
H	0.331161000000	-1.684307000000	-2.964908000000
H	0.992970000000	-2.870806000000	-4.125875000000
H	2.061849000000	-1.701321000000	-3.292676000000
C	0.860587000000	-7.058968000000	-1.345207000000
H	1.763223000000	-7.608612000000	-1.666180000000
H	0.095662000000	-7.210830000000	-2.122965000000

H	0.503895000000	-7.534212000000	-0.417941000000
C	2.239506000000	-3.256440000000	1.691950000000
H	2.196942000000	-4.064206000000	2.438199000000
H	1.556102000000	-2.454526000000	2.003402000000
H	3.245384000000	-2.816064000000	1.705286000000
C	-2.030944000000	3.086609000000	0.090992000000
C	-1.767525000000	3.976027000000	1.153951000000
C	-1.617067000000	5.343053000000	0.881486000000
H	-1.426593000000	6.031125000000	1.711418000000
C	-1.697983000000	5.849983000000	-0.422155000000
C	-1.915792000000	4.942567000000	-1.467958000000
H	-1.959093000000	5.314554000000	-2.496765000000
C	-2.077266000000	3.569463000000	-1.235026000000
C	-1.641357000000	3.452714000000	2.564822000000
H	-1.543912000000	4.270192000000	3.294417000000
H	-0.764509000000	2.794034000000	2.661969000000
H	-2.513068000000	2.837789000000	2.836738000000
C	-1.573613000000	7.330179000000	-0.692030000000
H	-1.122446000000	7.526501000000	-1.677440000000
H	-0.958679000000	7.832183000000	0.071118000000
H	-2.563929000000	7.818968000000	-0.685526000000
C	-2.304840000000	2.620187000000	-2.387861000000
H	-3.304477000000	2.166648000000	-2.336223000000
H	-1.598382000000	1.779518000000	-2.357336000000
H	-2.200737000000	3.129860000000	-3.357590000000

Supplementary Table 3. Cartesian coordinates (level of theory: B3-LYP/def2-SVP with GD3 dispersion correction) for Li[$(OC)_5$W(C(O)Ph)].

W	-1.029685000000	1.743698000000	0.401832000000
C	0.087324000000	-1.122349000000	-1.096253000000
C	-0.281298000000	-2.383486000000	-1.604902000000
C	0.430293000000	-2.964061000000	-2.653818000000
C	1.545477000000	-2.308749000000	-3.191096000000
C	1.937506000000	-1.068086000000	-2.678623000000
C	1.204871000000	-0.474493000000	-1.648613000000
C	-0.710289000000	-0.515344000000	0.025258000000
O	-1.281118000000	-1.376195000000	0.759974000000
Li	-1.939889000000	-1.304499000000	2.378428000000
C	-1.392717000000	3.741222000000	0.791392000000
O	-1.602325000000	4.848760000000	1.028932000000
O	-0.454675000000	2.294879000000	-2.760256000000
C	0.976714000000	1.965142000000	0.915422000000
C	-0.639647000000	2.108458000000	-1.645558000000
C	-1.495500000000	1.096518000000	2.229458000000
C	-3.017653000000	1.417174000000	-0.106522000000
O	-1.814893000000	0.456872000000	3.174939000000
O	2.086260000000	2.064165000000	1.200692000000
O	-4.118612000000	1.190731000000	-0.352193000000
H	-1.142693000000	-2.889778000000	-1.165211000000
H	0.120583000000	-3.933094000000	-3.054333000000
H	2.108245000000	-2.765892000000	-4.009252000000
H	2.813636000000	-0.557784000000	-3.086306000000
H	1.515212000000	0.492842000000	-1.252231000000

Supplementary Table 4. Cartesian coordinates (level of theory: B3-LYP/def2-SVP with GD3 dispersion correction) for borylene **3b.**

B	0.199001000000	0.294054000000	-0.240252000000
H	-0.617362000000	1.180053000000	-0.154893000000
C	1.600422000000	0.751362000000	-0.090122000000
O	1.899990000000	2.069893000000	0.103615000000
Si	1.072738000000	3.549191000000	-0.005120000000
C	0.402102000000	3.789835000000	-1.743360000000
H	1.159321000000	3.509079000000	-2.493473000000
H	0.136287000000	4.847875000000	-1.906590000000
H	-0.494491000000	3.180166000000	-1.917321000000
C	2.440929000000	4.790731000000	0.341021000000
H	2.880884000000	4.618392000000	1.336802000000
H	2.058023000000	5.824480000000	0.309911000000
H	3.248850000000	4.700914000000	-0.403100000000
C	-0.279518000000	3.726976000000	1.289863000000
H	-1.132152000000	3.059868000000	1.097451000000
H	-0.645983000000	4.768044000000	1.292404000000
H	0.109326000000	3.505459000000	2.296520000000
N	-1.724956000000	-1.307476000000	-0.168649000000
C	-0.393572000000	-1.109792000000	-0.360408000000
C	0.231717000000	-2.470345000000	-0.708981000000
C	-1.004258000000	-3.358732000000	-1.004442000000
H	-1.212029000000	-3.339803000000	-2.085916000000
H	-0.842612000000	-4.409300000000	-0.721926000000
C	-2.192200000000	-2.733806000000	-0.261101000000
C	1.127124000000	-2.398314000000	-1.955985000000
H	2.050254000000	-1.845060000000	-1.753306000000
H	0.603791000000	-1.909473000000	-2.792293000000
H	1.405755000000	-3.417777000000	-2.271694000000
C	1.054267000000	-3.033687000000	0.469654000000
H	0.487775000000	-3.034102000000	1.411545000000
H	1.968146000000	-2.450087000000	0.622479000000
H	1.349721000000	-4.072754000000	0.250311000000
C	-3.505570000000	-2.887285000000	-1.038757000000
H	-3.797920000000	-3.948329000000	-1.036105000000
H	-3.407706000000	-2.573443000000	-2.084573000000
H	-4.318618000000	-2.309944000000	-0.574359000000
C	-2.408156000000	-3.339599000000	1.137666000000
H	-2.689605000000	-4.398928000000	1.038303000000
H	-3.222605000000	-2.821013000000	1.661401000000
H	-1.507192000000	-3.286417000000	1.760254000000
C	-2.631663000000	-0.244745000000	0.182336000000
C	-3.332034000000	0.417413000000	-0.854695000000
C	-4.220953000000	1.443189000000	-0.506422000000
H	-4.767158000000	1.969388000000	-1.292372000000
C	-4.402602000000	1.820324000000	0.822656000000
H	-5.100519000000	2.622826000000	1.074992000000
C	-3.670718000000	1.189442000000	1.825599000000
H	-3.788518000000	1.517266000000	2.860872000000
C	-2.764936000000	0.159275000000	1.532006000000
C	-3.083987000000	0.123319000000	-2.331705000000
H	-2.480710000000	-0.790851000000	-2.395743000000

C	-2.242341000000	1.237497000000	-2.976734000000
H	-2.755048000000	2.211526000000	-2.916552000000
H	-2.059338000000	1.016000000000	-4.041144000000
H	-1.271727000000	1.325405000000	-2.470909000000
C	-4.383475000000	-0.110783000000	-3.118275000000
H	-5.016340000000	-0.876880000000	-2.645234000000
H	-4.155458000000	-0.439447000000	-4.145188000000
H	-4.980795000000	0.811548000000	-3.198326000000
C	-1.908686000000	-0.402884000000	2.663360000000
H	-1.299514000000	-1.217181000000	2.253994000000
C	-2.752205000000	-0.970648000000	3.816884000000
H	-2.103237000000	-1.457578000000	4.562809000000
H	-3.486353000000	-1.712372000000	3.468226000000
H	-3.308419000000	-0.174602000000	4.337481000000
C	-0.924596000000	0.658742000000	3.186393000000
H	-1.457262000000	1.522095000000	3.616529000000
H	-0.278113000000	1.018982000000	2.376232000000
H	-0.283721000000	0.230429000000	3.974065000000
C	2.886330000000	-0.008076000000	-0.036854000000
C	3.350470000000	-0.544267000000	1.183415000000
C	4.573429000000	-1.228407000000	1.207143000000
H	4.925451000000	-1.651051000000	2.153587000000
C	5.360614000000	-1.375694000000	0.058039000000
C	4.910410000000	-0.782202000000	-1.128273000000
H	5.527261000000	-0.854090000000	-2.029806000000
C	3.696940000000	-0.085194000000	-1.190724000000
C	2.549352000000	-0.361754000000	2.450040000000
H	3.060939000000	-0.803340000000	3.318189000000
H	1.551362000000	-0.815290000000	2.362566000000
H	2.381619000000	0.707920000000	2.651651000000
C	6.649749000000	-2.160520000000	0.093522000000
H	7.116141000000	-2.130212000000	1.090615000000
H	7.379056000000	-1.776215000000	-0.636700000000
H	6.473599000000	-3.223083000000	-0.151503000000
C	3.270194000000	0.604200000000	-2.464070000000
H	3.937813000000	0.358862000000	-3.303255000000
H	3.276152000000	1.697618000000	-2.324901000000
H	2.238947000000	0.335219000000	-2.739582000000

Suppplementary methods

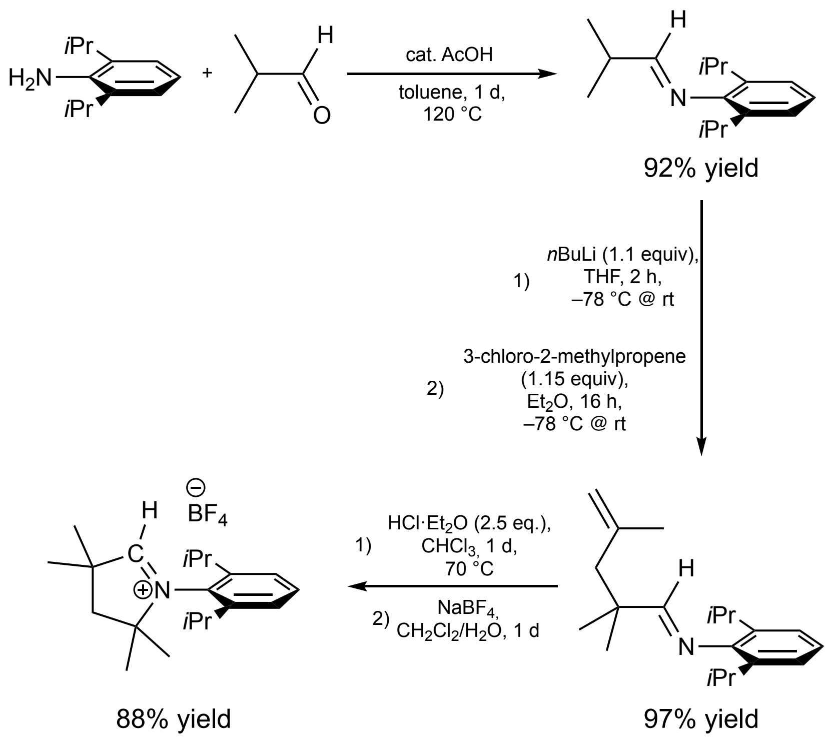

Supplementary Fig. 29. Synthesis of CAAC·HBF_4 based on a published procedure[1-3].

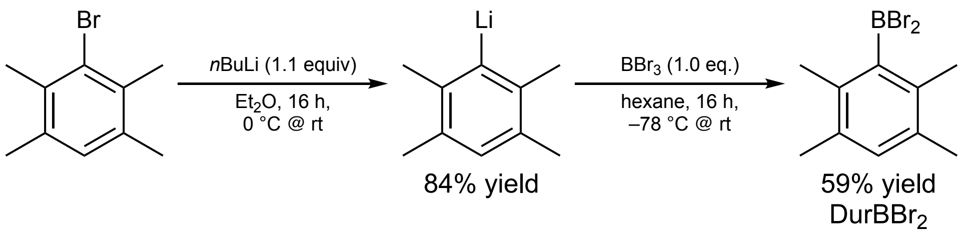

Supplementary Fig. 30. Synthesis of $DurBBr_2$ based on a published procedure[4,5].

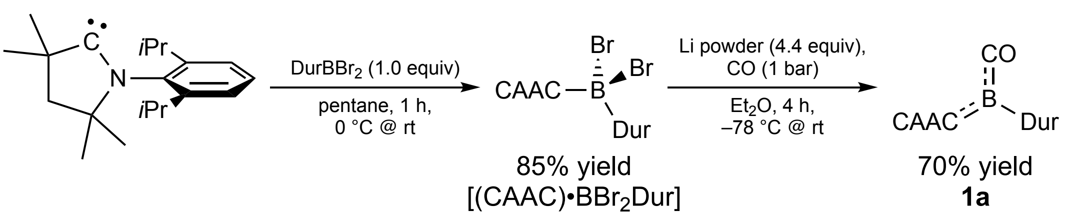

Supplementary Fig. 31. Synthesis of compound **1a** based on a published procedure[6].

Synthesis of [(CAAC)·BBr_2Dur] following a published procedure[6]. Pentane was rapidly added by cannula to a mixture of compound $DurBBr_2$ (1.0 equiv) and CAAC (1.0 equiv) at 0 °C with vigorous stirring. The reaction mixture was then allowed to warm to room temperature and stirred for one hour. A white precipitate was subsequently separated by centrifugation and

filtration from the resulting pink solution, and was washed twice with pentane to afford [(**CAAC)·BBr$_2$Dur]** as a white powder.

Synthesis of [(CAAC)·BDur(CO)] (1a) following a published procedure[6]. (CAAC)BBr$_2$Dur (1.50 g, 2.55 mmol) and lithium sand (77.7 mg, 11.2 mmol) were suspended in 100 mL of diethyl ether. The reaction mixture was stirred under an atmosphere of CO at room temperature for 4 h. The suspension was filtered all volatiles were removed *in vacuo* and the solid residue was recrystallised from benzene at room temperature, yielding **1a** as an orange solid in 70% yield (815 mg, 1.78 mmol).

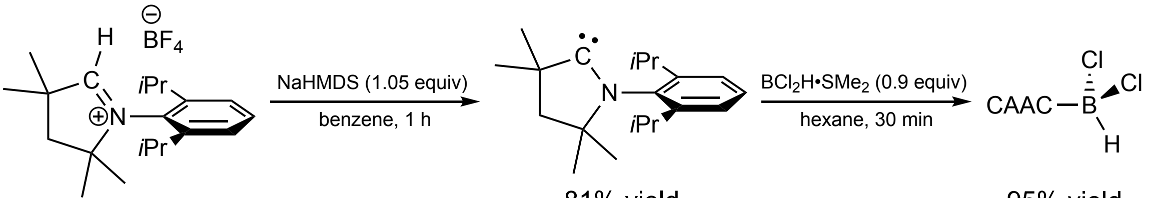

81% yield
CAAC

95% yield
[(CAAC)•BCl$_2$H]

Supplementary Fig. 32. Deprotonation of CAAC·HBF$_4$ and synthesis of [(CAAC)·BCl$_2$H] following a published procedure[3,7].

Synthesis of [(CAAC)·BCl$_2$H] following a published procedure[7]. CAAC (1.1 equiv) was dissolved in toluene and added to a stirred solution of commercial [(SMe$_2$)·BCl$_2$H] (1.0 equiv) in benzene. The reaction mixture was briefly warmed and a colourless solid crystallized upon cooling to room temperature. After allowing to sit for 1 d at room temperature, the solid residue was filtered and dried in vacuo. NMR data of the colorless solid, however, indicated the presence of a 4:1 mixture of [(CAAC)·BCl$_2$H] and [(CAAC)·BCl$_3$]. Note: For subsequent reduction to diborene [B$_2$H$_2$(CAAC)$_2$] no further purification was necessary.

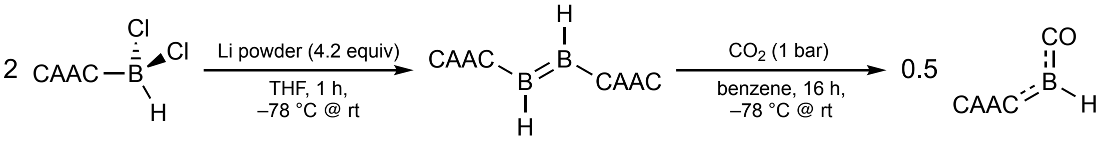

72% yield
[B$_2$H$_2$(CAAC)$_2$]

78% yield
1b

Supplementary Fig. 33. Synthesis of compound **1b** following a published procedure[7].

Synthesis of [B$_2$H$_2$(CAAC)$_2$] following a published procedure[7]. [(CAAC)·BCl$_2$H] (1.0 equiv) was dissolved in THF, the mixture was then cooled to –78 °C and transferred to a stirred suspension of elemental lithium powder (2.1 equiv) in THF at –78 °C. While stirring, the mixture was slowly warmed to room temperature over 1 h, whereupon it turned deep blue, and

the solvent was removed under reduced pressure. The residue was extracted with pentane and [B_2H_2(CAAC)$_2$] was obtained by removing the solvent of the extract under reduced pressure, recrystallization from a slowly evaporating benzene solution and washing of the crystalline material with benzene.

Synthesis of [(CAAC)·BH(CO)] (1b) following a published procedure[8]. A solution of B_2H_2(CAAC)$_2$ (972 mg, 1.63 mmol) in benzene (25 mL) was cooled to –78 °C and the argon atmosphere was replaced with CO_2 (1 bar). The reaction mixture was slowly warmed to room temperature whereupon within 4 h at rt a color change from blue to orange was observed. Subsequently the solvent was evaporated under vacuum. The residue was sublimed at 90-130 °C under high vacuum to yield **1b** as an orange solid. Single crystals suitable for X-ray diffraction analysis were obtained by evaporation of a saturated benzene solution at rt in the glovebox. Yield: 399 mg (1.27 mmol, 78%) as an orange solid.

Supplementary References

1. Jazzar, R. et al., Intramolecular "Hydroiminiumation" of Alkenes: Application to the Synthesis of Conjugate Acids of Cyclic Alkyl Amino Carbenes (CAACs). *Angew. Chem., Int. Ed.* **46**, 2899-2902 (2007).
2. Jazzar, R., Bourg, J.-B., Dewhurst, R. D., Donnadieu, B. & Bertrand G. Intramolecular "Hydroiminiumation and -amidiniumation" of Alkenes: A Convenient, Flexible, and Scalable Route to Cyclic Iminium and Imidazolinium Salts. *J. Org. Chem.* **72**, 3492-3499 (2007).
3. Pichon, D., et al. The debut of chiral cyclic (alkyl)(amino)carbenes (CAACs) in enantioselective catalysis. *Chem. Sci.* **10**, 7807-7811 (2019).
4. Auner, N., Salzer, A., Herrmann, W. A. & Brauer, G. *Synthetic Methods of Organometallic and Inorganic Chemistry, Group 1, 2, 13 and 14, Vol. 2*, Thieme (1996).
5. Braunschweig, H., Ye, Q. & Radacki, K. High yield synthesis of a neutral and carbonyl-rich terminal arylborylene complex. *Chem. Commun.* **48**, 2701-2703 (2012).
6. M.-A. Légaré, et al. Nitrogen fixation and reduction at boron. *Science* **359**, 896-900 (2018).
7. M. Arrowsmith, et al. Direct access to a cAAC-supported dihydrodiborene and its dianion. *Chem. Commun.* **54**, 4669-4672 (2018).
8. A. Stoy, et al. Evidence for Borylene Carbonyl (LHB=C=O) and Base-Stabilized (LHB=O) and Base-Free Oxoborane (RB≡O) Intermediates in the Reactions of Diborenes with CO_2. *J. Am. Chem. Soc.* **144,** 3376-3380 (2022).
